# SemNet Explorer: An Evidence-Grounded Knowledge Graph–LLM Framework for Multi-Scale Mechanistic Reporting Across Biomedical Domains

**DOI:** 10.3390/bdcc10060171

**Published:** 2026-05-25

**Authors:** Xin He, David Camacho, Lama Moukheiber, Meghna Iyer, Benjamin Zhao, Christophe Ye, Batuhan Nursal, Xinyu Guo, Albert J. B. Lee, Cassie S. Mitchell

**Affiliations:** 1 Laboratory for Pathology Dynamics, Georgia Institute of Technology, Emory University School of Medicine, Atlanta, GA 30332, USA; 2 Department of Mathematics, Brigham Young University, Provo, UT 84602, USA; 3 Center for Machine Learning at Georgia Tech, Georgia Institute of Technology, Atlanta, GA 30332, USA

**Keywords:** knowledge graph, large language models, graph-based reasoning, SemNet 2.0, mechanistic reporting, multimorbidity, comparative pathophysiology, drug repurposing, adverse event analysis, evidence grounding, adaptive prompting, process enrichment, Venn decomposition

## Abstract

**Background:**

Mechanistic reporting from large-scale biomedical knowledge graphs remains challenging, particularly when integrating structured graph evidence with large language model (LLM)–based explanation in a reproducible and auditable manner. Existing approaches either rely on manual synthesis of graph-derived results or generate unconstrained narratives that lack traceability to underlying evidence.

**Methods:**

We present **SemNet Explorer**, an evidence-grounded knowledge graph–LLM unified framework for automated mechanistic reporting across biomedical domains using SemNet 2.0, a PubMed-scale heterogeneous knowledge graph. Given a set of target concepts and a selected semantic layer, the framework organizes graph-derived evidence into structured regions and generates two complementary report types: global reports for process-level mechanisms and anchor-centric reports for localized mediator-based explanations. A central methodological contribution is an ablation-derived adaptive grounding policy: we systematically compare alternative evidence-integration strategies across report types, semantic layers, and region structures, and use the resulting preferences to guide prompt selection in the deployed system.

**Results:**

SemNet Explorer produces stable region decompositions and interpretable report scaffolds across molecular (AAPP), disease-level (DSYN), and pharmacologic (PHSU) representations. For global reports, explicit evidence grounding improves expression quality more consistently than content accuracy, with benefits dependent on evidence density and semantic abstraction. In contrast, anchor-centric reports show consistent improvements in both content and expression under stronger, mediator-constrained prompting. These findings are supported by both pairwise ablation comparisons and absolute score analyses.

**Conclusions:**

SemNet Explorer establishes a generalizable unified framework and interactive platform for transforming knowledge graph evidence into reproducible mechanistic narratives across biomedical domains, including multimorbidity analysis, comparative pathophysiology, drug repurposing, and adverse event discovery. The results demonstrate that effective knowledge graph–LLM integration requires adaptive, context-dependent evidence grounding rather than fixed prompting strategies.

## 1. Introduction

Alzheimer’s disease (AD), amyotrophic lateral sclerosis (ALS), and frontotemporal dementia (FTD) are clinically distinct neurodegenerative disorders, but they also show substantial overlap in pathology, genetics, and molecular mechanism [1-10]. Protein aggregation provides one of the clearest points of convergence: amyloid-*β* and tau are central in AD, TDP-43 and SOD1 are prominent in ALS, and tau- or TDP-43-positive pathology is common in FTD [7,8]. At the same time, overlap does not imply identity; disease-specific modifiers, selective vulnerability, and higher-level systems effects still shape distinct clinical phenotypes [11,12]. The comparative task is therefore twofold: to explain both cross-disease *comorbidity* and disease-specific *divergence*, and to do so across molecular, syndrome-level, and pharmacologic representations.

Heterogeneous knowledge graphs provide a natural framework for this problem because they can organize large-scale biomedical concepts and relations in a form that supports structured comparison. Literature-based discovery, beginning with Swanson’s work on hidden cross-literature connections, established the broader logic of using distributed biomedical evidence to surface non-obvious links [13]. SemNet and SemNet 2.0 extend this direction through a PubMed-scale UMLS-based semantic graph coupled with relatedness scoring for disease-centered concept ranking and hypothesis generation [14,15]. These approaches are consistent with broader efforts in biomedical knowledge graph construction and application, including large-scale integrative graphs and drug repurposing frameworks [16,17]. Related work has also explored embedding-based and graph-based learning methods to extract structured relationships and support downstream predictive modeling [18]. SemNet-enabled approaches have been applied across a range of biomedical problems, including disease mechanism discovery, drug repurposing, adverse event prediction, and multimorbidity analysis in settings such as chronic kidney disease, COVID-19, cancer, and neurodegenerative disorders [19-23], consistent with broader applications of knowledge graph–driven biomedical discovery and AI-assisted hypothesis generation [24].

Prior SemNet 2.0 studies on the AD–ALS–FTD triad showed that meaningful shared and disease-specific structure can indeed be recovered at multiple semantic layers, including **AAPP** (amino acids, peptides, and proteins), **DSYN** (diseases and syndromes), and **PHSU** (pharmacologic substances), but those analyses still mainly ended in ranked lists, category mappings, and manuscript-level interpretation rather than a reusable reporting workflow [11,12]. In practice, this interpretation step required substantial manual effort: analysts needed to inspect ranked node lists, map high-ranking concepts to biological or process categories, compare patterns across disease combinations, and iteratively synthesize these findings into coherent mechanistic narratives. Depending on the scope of the analysis, this process typically required several hours for a single comparison and up to one to two days for multi-disease, multi-layer studies. This labor-intensive workflow limited both the scale of hypothesis exploration and the reproducibility of reported conclusions, as the final interpretation depended heavily on manual synthesis.

Large language models (LLMs) create a complementary opportunity because they can convert structured evidence into readable biomedical narratives. However, recent reviews in biomedicine and healthcare also emphasize that unconstrained LLM outputs are difficult to verify, trace, and reproduce, especially in high-stakes scientific or medical settings [25]. This has motivated retrieval-augmented generation (RAG) and related grounding strategies, which explicitly attach external evidence to generation rather than relying only on parametric recall [26,27]. More recent GraphRAG and knowledge-graph-grounded generation studies further suggest that graph structure can preserve relations, support multi-hop evidence integration, and improve evidence-aware reasoning beyond flat text retrieval [28-30]. These approaches highlight the growing importance of integrating structured knowledge representations with generative models for scientific reasoning and explanation [31]. Yet most of this literature is framed around question answering, medical response generation, or hypothesis generation rather than comparative mechanistic reporting across shared and disease-specific disease regions.

SemNet Explorer is related to GraphRAG in that both use graph-structured evidence to constrain LLM generation. However, the retrieval objective and evidence organization differ. Typical GraphRAG pipelines retrieve graph neighborhoods, communities, paths, or text-linked graph summaries to answer a user query. SemNet Explorer instead begins with a fixed comparative disease setting and constructs a reporting scaffold before generation: anchors are assigned to Venn regions, ranked by multi-signal graph evidence, mapped to process/category inventories, and optionally expanded into mediator-level drill-down evidence. Thus, the graph is not used only as a retrieval backend, but as a task-specific evidence organizer that separates shared-core, pairwise, and disease-specific mechanisms before prompting. This distinction motivates the adaptive prompting strategy: because evidence density, semantic abstraction, and report granularity vary across regions and layers, the optimal level of evidence grounding should also vary rather than remain fixed.

This paper addresses that gap through **SemNet Explorer**, a unified interactive framework built on SemNet 2.0 for automated, evidence-grounded mechanistic reporting across multiple diseases and biomedical contexts. The aim is not simply to retrieve relevant graph evidence, but to replace labor-intensive manual interpretation with a structured, automated pipeline that organizes evidence into explicit shared and disease-specific regions and generates two complementary forms of report: process-level reports for regional biological patterns and anchor-level reports for specific explanatory nodes. A second focus is how evidence should be introduced into LLM generation. Rather than treating prompt design as fixed, we compare alternative evidence-integration styles across report types, semantic layers, and Venn regions, and use the resulting ablation patterns to derive an adaptive prompting policy for deployment. The contribution is therefore not another static analysis of AD, ALS, and FTD, but a reusable framework for producing auditable reports of disease *comorbidity* and *divergence* from structured graph evidence.

Although we focus on the AD–ALS–FTD triad as a well-characterized benchmark with prior manual analyses, the framework is general and supports multiple biomedical use cases, including multimorbidity analysis, comparative disease modeling, drug target and repurposing evaluation, and adverse event analysis.

## 2. Materials and Methods

### 2.1. System Overview

**Figure 1.**
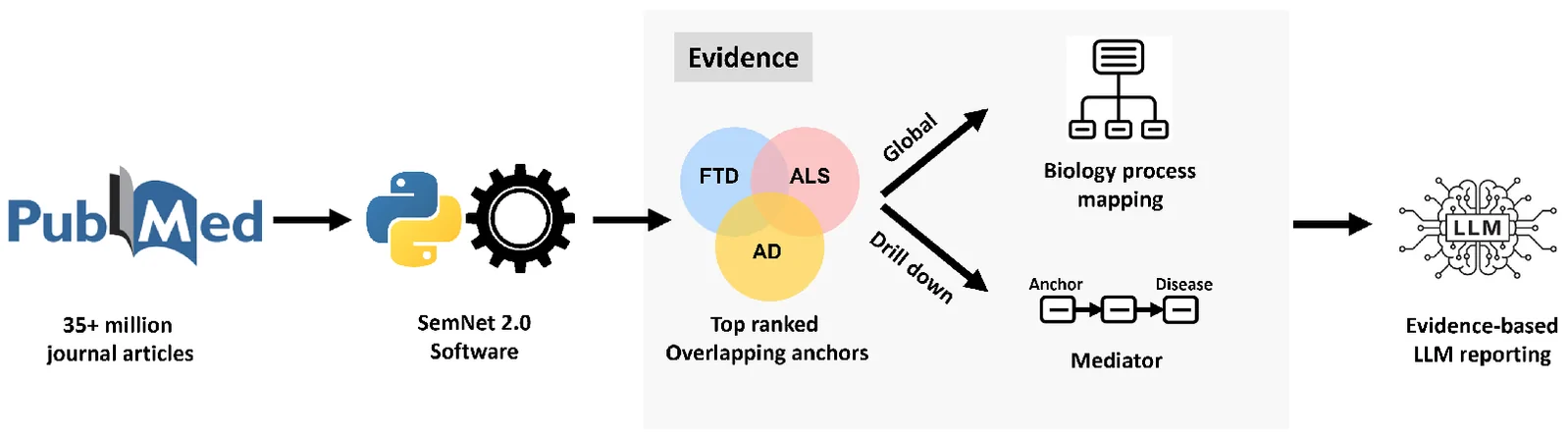
Overall workflow of the proposed SemNet 2.0–based interactive, unified framework for evidence-grounded tri-disease comparative reporting. Ranked overlapping anchors are derived from SemNet 2.0 and organized into (i) a global process/category scaffold and (ii) a drill-down mediator-chain view (anchor–mediator–disease). These evidence views support structured LLM report generation under explicit evidence constraints.

**SemNet Explorer** is an interactive, unified framework for evidence-grounded mechanistic reporting across disease triads. Given three diseases (*d*_1_, *d*_2_, *d*_3_) and a selected semantic layer *T*, the system converts graph-derived evidence into structured, evidence-grounded reports through the following stages: (i) anchor evidence discovery, region decomposition, and anchor ranking; (ii) aggregation of anchor evidence into process-level global evidence; (iii) drill-down of anchor evidence into mediator-level local evidence; and (iv) ablation-based derivation and deployment of an adaptive evidence-injection policy for LLM report generation and judge-based audit. Figure 1 summarizes this end-to-end workflow. Conceptually, this pipeline defines a structured transformation from graph-derived relational evidence to constrained natural language explanation, with explicit intermediate representations that preserve traceability between source evidence and generated claims. While demonstrated here on disease triads, the same pipeline applies to broader biomedical contexts, including drug-centric and cross-domain analyses, by varying the input node types and target concepts.

**Figure 2.**
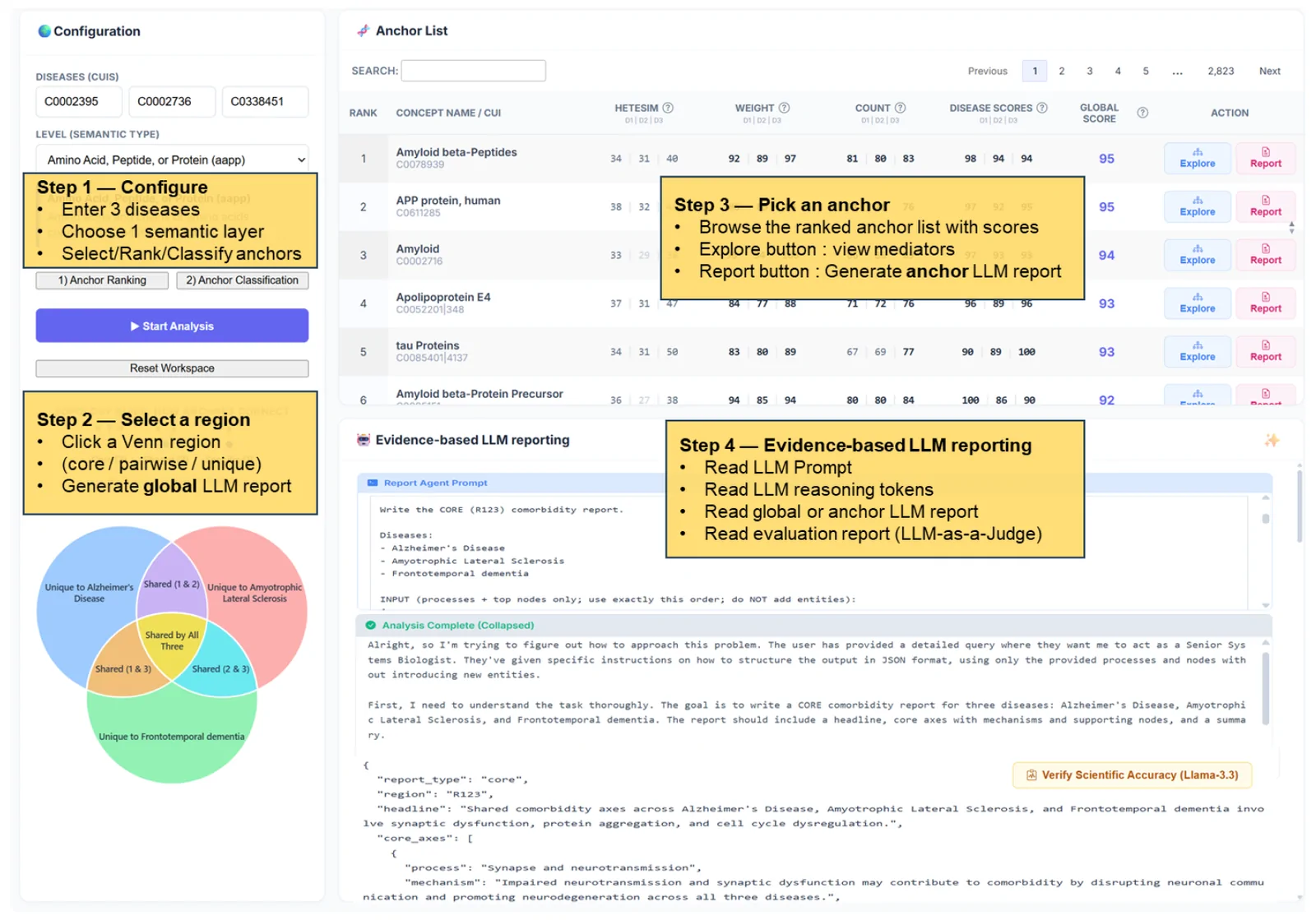
Interface walkthrough for using the SemNet Explorer platform. Step 1: configure a disease triad and semantic layer. Step 2: select a Venn region to define a global reporting objective. Step 3: browse ranked anchors and use *Explore* to inspect mediator evidence or *Report* to generate an anchor-centric report. Step 4: view evidence-grounded LLM outputs and optionally run rubric-based verification.

The platform is implemented as a Flask web application. Users interact through tri-disease input, semantic-layer selection, Venn-region selection, and anchor drill-down. Report generation is streamed to the interface and saved as JSON artifacts. A step-by-step interface walkthrough is shown in Figure 2.

SemNet Explorer is built on **SemNet 2.0**, a PubMed-scale, literature-derived heterogeneous biomedical knowledge graph [14,15]. SemNet 2.0 is constructed from semantic predications mined from PubMed abstracts and represented as a directed graph in which nodes are Unified Medical Language System (UMLS) concepts and edges are semantically typed predicates [14,15]. The graph spans 133 semantic types and 54 relation types, allowing traversal across molecular, pharmacologic, disease, and clinical layers within one representation [15]. To quantify disease-conditioned semantic relatedness, the platform also uses SemNet 2.0 metapath-based similarity scores computed with **HeteSim**; these signals are incorporated into anchor ranking [15,32].

### 2.2. Manual Versus Automated Workflow Comparison

To contextualize the practical impact of the proposed framework, we contrast the workflow required for prior SemNet-based comparative studies with the automated pipeline implemented in SemNet Explorer. In the manual setting, analysts typically (i) inspect ranked anchor lists across semantic layers, (ii) map high-ranking concepts to biological or process categories, (iii) compare category distributions across disease combinations, and (iv) iteratively synthesize mechanistic explanations into narrative form. Based on prior study experience, this process typically requires several hours for a single disease comparison and up to one to two days for multi-disease, multi-layer comparative analysis.

In contrast, SemNet Explorer integrates anchor discovery, region decomposition, process enrichment, and report generation into a single pipeline. Once inputs are specified, report generation—including evidence structuring and LLM-based synthesis—occurs within seconds. Importantly, the automated pipeline also preserves intermediate evidence representations and structured outputs, enabling reproducibility, auditability, and consistent report generation that are difficult to achieve in manual workflows.

### 2.3. Anchor Discovery, Venn Region Decomposition, and Ranking

We use the term *anchor* to denote a candidate concept in the selected semantic layer that serves as a focal explanatory unit linking one or more diseases through compact graph structure. Given a disease triad (*d*_1_, *d*_2_, *d*_3_) and semantic layer *T*, the platform discovers candidate anchors *a* ∈ *T* by bounded two-hop enumeration around each disease. The default pattern is

a→m→d,

where *m* is an intermediate concept and *d* is a disease endpoint. In the present study, the three diseases are Alzheimer’s disease (AD), amyotrophic lateral sclerosis (ALS), and frontotemporal dementia (FTD), treated as *d*_1_, *d*_2_, and *d*_3_, respectively. Candidate anchors are taken from the union of per-disease two-hop reachable nodes in type *T*. Immediate backtracking to the previously visited node is disallowed during traversal.

These two-hop connections are then used to assign each anchor to a disease-membership region. An anchor is considered connected to disease *d_i_* if at least one valid chain *a* → *m* → *d_i_* exists. In the AD–ALS–FTD setting, *R*_123_ contains anchors connected to all three diseases; *R*_12_, *R*_13_, and *R*_23_ contain anchors connected to exactly the AD–ALS, AD–FTD, and ALS–FTD pairs, respectively; and *R*_1_, *R*_2_, and *R*_3_ contain anchors connected only to AD, only to ALS, and only to FTD. Thus, *R*_123_ represents shared tri-disease structure, *R*_12_, *R*_13_, *R*_23_ represent pairwise overlaps, and *R*_1_, *R*_2_, *R*_3_ represent disease-specific signatures. This decomposition provides the routing structure for downstream reporting.

Within each region, anchors are ranked using three complementary evidence signals. **Weight/strength** measures evidence intensity by aggregating literature-supported two-hop chain strengths between an anchor and a disease. **HeteSim** measures semantic specificity under metapath-conditioned relatedness. **Mediator count** measures evidence breadth by counting distinct mediators that support at least one valid chain to the disease.

For each disease *d_i_*, these signals are transformed to a common nonnegative scale. Log compression is applied to count- or strength-like quantities when needed, and each component is normalized within the candidate set for the current run. This yields disease-specific scores *H_i_*(*a*), *W_i_*(*a*), and *C_i_*(*a*), which are combined by harmonic mean,

$$S_{i}(a)=\text{HM}(H_{i}(a),W_{i}(a),C_{i}(a)).$$

This formulation favors anchors that are simultaneously specific, strongly supported, and broadly connected, rather than dominant in only one dimension.

For region-conditioned ranking, the disease-specific scores are aggregated across the diseases that define the region using the same harmonic-mean principle. This favors anchors with balanced support within the selected region and downweights cases driven mainly by one dominant disease connection. The resulting ranked anchor lists serve as the starting point for both region-level process summarization and anchor-level mechanistic drill-down.

### 2.4. Anchor-to-Biological Process Mapping and Region-Level Enrichment

The ranked anchor lists are next converted into a compact global evidence view through anchor-to-process mapping. Following prior SemNet comparative studies, high-ranking nodes are mapped to predefined biological or mechanistic process categories to improve interpretability and support cross-region comparison [11,12]. Specifically, anchors are classified into closed-set biological/process categories using two ontology tracks adopted from prior SemNet 2.0 comparative work: a *micro-mechanistic* ontology for molecular and cellular layers and a *macro-systemic* ontology for higher-level disease and pharmacologic layers. Classification is implemented with an instruction-tuned LLM (*Qwen2.5-7B-Instruct*) as a strict single-label selection task. For each anchor, the model must choose exactly one category from the provided list. The process/category inventories were not newly introduced in this study. The micro-mechanistic ontology used for AAPP and related molecular layers was adopted from prior SemNet 2.0 work on AD–ALS–FTD molecular overlap, where high-ranking AAPP nodes were mapped to biological processes using supervised human review and external LLM-assisted classification. The macro-systemic ontology used for DSYN and PHSU layers was adopted from prior SemNet 2.0 multimorbidity work, where DSYN nodes were mapped to 13 mechanistically interpretable categories and reviewed by three human evaluators through full-text inspection, with high inter-rater reliability (Cohen’s *κ* = 0.88). In the present study, the LLM-based classifier is therefore used to apply these predefined category inventories consistently to newly generated region-level anchor lists, rather than to define a new ontology.

For each region *r*, let *c*_*r,p*_ denote the number of anchors assigned to process/category *p*, and let $N_{r}=\sum_{p}c_{r,p}$ denote the number of classified anchors in that region. We estimate a background frequency *p*_bg_(*p*) from the union of classified anchors across all regions. Region-level enrichment is then summarized by

$$z_{\text{enrich}}(r,p)=\frac{c_{r,p}-N_{r}\;p_{\text{bg}}(p)}{\sqrt{N_{r}\;p_{\text{bg}}(p)\;(1-p_{\text{bg}}(p))}},$$

which compares the observed count against the expected count under the layer-specific background. Categories are then labeled as enriched, depleted, or neutral using a fixed *z*-threshold.

The enrichment output is compiled into compact region payloads for global reporting. Each payload contains the top enriched processes together with short anchor evidence lists. These region-level process summaries define the evidence blocks used for global reports and provide a process-level view of comorbidity and divergence that is more compact and more interpretable than long ranked anchor lists alone.

### 2.5. Mediator Evidence and Drill-Down Structure

In addition to process-level aggregation, the platform preserves a local drill-down evidence view centered on mediators. For each anchor, we enumerate fixed-length two-hop chains of the form *a* → *m* → *d*, where *m* is an intermediate mediator and *d* is a disease endpoint. The mediators linked to each anchor are collected separately for AD, ALS, and FTD, with duplicate mediators removed within each disease-specific set.

These mediator sets are then grouped according to whether a mediator supports more than one disease path or only a single disease path, yielding shared versus disease-specific local evidence for the selected anchor. This mediator-level evidence is used directly in anchor-centric report generation to explain either why an anchor supports multimorbidity across the tri-disease core or why it is more characteristic of a disease-specific region.

In this way, the local mediator view complements the global process scaffold: anchor evidence is summarized upward into biological processes for region-level reports, while the same anchor can also be expanded downward into mediator evidence for anchor-centric drill-down.

### 2.6. Evidence-Grounded Report Formulation

Once region-level and mediator-level evidence structures are available, the platform generates two complementary report families: **global region reports** and **anchor-centric reports**. In both cases, the report objective is determined by the selected region, the selected semantic layer, and the available graph-derived evidence. Outputs are constrained to strict JSON schemas so that reports can be streamed to the interface, saved as structured artifacts, and optionally reviewed downstream. This formulation enforces a consistent mapping between structured evidence inputs and generated outputs, supporting reproducibility and auditability of the reporting process.

**Global region reports** operate on the region payloads produced by anchor-to-process mapping and region-level enrichment. When a user selects a region, the system generates a region-level synthesis report with a region-specific objective. For the shared core *R*_123_, the task is to explain dominant mechanisms shared across AD, ALS, and FTD. For pairwise regions *R_ij_*, the task is to explain mechanisms linking the selected disease pair while contrasting the third disease. For unique regions *R_i_*, the task is to explain biological signatures that are more characteristic of the target disease than of the other two. The prompt includes enriched processes and representative anchors as structured evidence blocks, and the output is returned as JSON with report-level fields such as report_type, region, headline, and summary, plus per-axis process/mechanism entries.

**Anchor-centric reports** operate on a selected anchor together with its local mediator evidence. In the deployed application, anchor reports are generated for the shared core (*R*_123_) and for disease-specific regions (*R*_1_, *R*_2_, *R*_3_). For shared-region anchors, the task is to explain why the anchor may support multimorbidity across all three diseases. For unique-region anchors, the task is to explain why the anchor appears more characteristic of the target disease than of the other two. These outputs are also returned as strict JSON, centered on report_type, region, anchor, and a single mechanism field.

In addition to region objectives, report wording is lightly adapted by semantic layer. Molecular layers emphasize pathways, metabolism, proteostasis, signaling, immune activation, and cellular dysfunction. Pharmacologic layers emphasize modulation, intervention, target relevance, and disease-biased therapeutic alignment. Clinical layers emphasize phenotype coupling, syndrome progression, shared burden, and disease-biased syndrome structure.

### 2.7. Evidence-Injection Ablation, Adaptive Prompt Policy, and Judge Evaluation

Because the platform relies on evidence-grounded LLM generation, a central methodological question is how strongly graph-derived evidence should be injected into the prompt. We therefore evaluated alternative evidence-injection policies before deployment. The evaluation covered three semantic layers—**AAPP**, **PHSU**, and **DSYN**—and four regions: the shared tri-disease core (*R*_123_) and the three disease-specific regions (*R*_1_, *R*_2_, *R*_3_).

The adaptive prompting strategy follows from an evidence–task alignment principle. Stronger evidence injection is expected to help when the provided graph evidence is specific, sufficient, and closely matched to the reporting objective, because the model can use concrete anchors or mediators to support bounded mechanistic claims. Conversely, strong grounding can degrade content when evidence is sparse, overly abstract, or weakly aligned with the requested explanation, because the model may be forced to over-interpret a small or indirect evidence set. We therefore treat prompt strength as a function of report family, semantic layer, and region structure rather than as a globally fixed setting. Global reports aggregate process-level evidence across a region, whereas anchor-centric reports use local mediator evidence; AAPP, PHSU, and DSYN also differ in mechanistic specificity. These differences provide the rationale for deriving the final prompt policy empirically from matched ablation comparisons.

We selected these four regions for the ablation because the primary evaluation objective was to test the two most interpretable reporting targets: shared tri-disease mechanisms (*R*_123_) and single-disease divergence (*R*_1_, *R*_2_, *R*_3_). Pairwise regions such as *R*_12_ were retained in the region-decomposition, composition, and enrichment analyses, but they were not evaluated in the same depth in the prompt-ablation experiments.

We tested four prompt modes over 100 repeated runs. The **base** mode used only the task objective and minimal structured input, without explicit supporting evidence. The three evidence-grounded modes differed in how evidence was injected: **soft evidence**, in which supporting nodes or mediators were optional; **inline evidence constraints**, in which the model was required to use the provided evidence explicitly; and **claim-then-example**, in which the model first stated a concise mechanistic claim and then supported it with evidence examples.

For **global process reports**, we performed pairwise ablations between the base prompt and the three stronger evidence-grounded variants. The base condition received only the enriched process list and region objective, without process-specific anchor evidence. The evidence-grounded conditions used the same process scaffold but differed in how explicitly representative anchors were injected. For each node-type/region setting, an external judge LLM compared two reports generated under the same task and selected the better one on two dimensions. **Content accuracy** measured biological and mechanistic adequacy, including plausibility, correctness of biological roles/functions, and overall mechanistic coherence. **Expression quality** measured whether the explanation was concrete rather than generic, appropriately supported rather than vague, and properly bounded rather than exaggerated. Results were summarized as win rates,

$$\text{win rate}=\frac{\#(\text{test beats base})}{\#(\text{all pairwise comparisons})},$$

so that values above 0.5 indicate preference for the evidence-grounded variant over the baseline.

We applied the same design to **anchor-centric reports**. Here, the reporting unit was a single anchor together with its local mediator evidence. The base prompt used only the anchor, disease context, and region objective, whereas the evidence-grounded variants introduced mediator evidence with increasing levels of constraint and structure. In the shared core (*R*_123_), prompts were built from shared mediator evidence; in the unique regions (*R*_1_, *R*_2_, *R*_3_), they were built from target-disease-specific anchor–mediator evidence. Judge-based pairwise comparisons again used **content accuracy** and **expression quality**.

Pairwise comparisons show relative preference but not the absolute score profile of each mode. We therefore added a **single-report score-based validation** for both global and anchor reporting. For each of the 12 node-type/region settings (AAPP, PHSU, DSYN crossed with *R*_123_, *R*_1_, *R*_2_, *R*_3_), we scored all four prompt modes independently with the same judge-model family. For each report, the judge returned **content accuracy** and **expression quality** scores; results were aggregated by node type, region, and mode to obtain means and standard deviations over 100 repeated runs. These combined analyses were then used to derive the adaptive prompting policy adopted in the deployed application.

To strengthen statistical interpretation of repeated judge comparisons, we added post hoc confidence intervals and significance tests for win-rate summaries. For each pairwise comparison, we computed Wilson 95% confidence intervals for the test-mode win rate and exact two-sided binomial tests against a null win probability of 0.5. We interpreted a setting as a robust improvement only when the win rate was above 0.5, the Wilson lower confidence bound was above 0.5, the binomial test satisfied *p* < 0.05, and at least 20 comparisons were available. Settings with win rates above 0.5 but confidence intervals overlapping 0.5 were treated as directional improvements. Settings with fewer than 20 evaluated comparisons were reported as sparse descriptive cases rather than statistically robust evidence.

The final deployment policy was specified explicitly from the ablation summaries rather than inferred manually from the figures. For each report family, semantic layer, and region, the candidate evidence-grounded modes were ranked primarily by the content-accuracy win rate against the base prompt, with expression-quality win rate used as a secondary criterion when content scores were close. If the best evidence-grounded mode did not exceed the base-parity threshold of 0.5 for content accuracy, the policy fell back to the base prompt. Thus, the deployed policy follows a content-first, expression-second selection rule, while allowing fallback to the base prompt in settings where stronger grounding did not improve judged content. For global process reports, this rule was applied to the process-level ablation summaries; for anchor-centric reports, it was applied to the anchor-level mediator-grounding ablation summaries.

Additional implementation details for reproducibility are provided in Appendix B, including anchor traversal and filtering rules, score normalization, exact report-generation prompt templates with dynamic placeholders, judge prompt templates, decoding settings, JSON validation, adaptive policy selection, and anchor-to-process category inventories.

### 2.8. Retrospective Validation Using Prior Expert-Curated SemNet Studies

To complement the LLM-as-judge ablation analysis, we performed a retrospective validation analysis using previously published SemNet 2.0 case studies that had undergone manual expert interpretation and post-analysis. This validation was designed to assess whether SemNet Explorer could recover major expert-derived mechanistic findings across multiple biomedical application domains.

Five prior SemNet-based studies were selected because they span distinct biomedical discovery tasks: comparative pathophysiology, multimorbidity/comorbidity analysis, drug repurposing, adverse-event prediction, and mechanistic systems biology. These prior studies incorporated downstream biomedical or clinician-guided interpretation, including ontology grouping, mechanistic contextualization, and assessment of the biological relevance of highly ranked graph-derived concepts. They therefore provide expert-curated reference conclusions for retrospective comparison.

For each prior study, we identified one primary expert-derived mechanistic finding and, where applicable, one secondary supporting finding from the published results and discussion. SemNet Explorer was then run using comparable disease, drug, or pathway anchor concepts. Global reports were used when the prior finding referred to a region-level theme, such as shared disease mechanisms, therapeutic classes, adverse-event clusters, or broad signaling modules. Anchor-centric reports were used when the prior finding involved a specific drug, drug class, protein, or pathway node.

Recovery was assessed qualitatively at the level of major biological themes, mechanistic regions, pathway classes, therapeutic categories, and systems-level disease architectures. A finding was considered recovered when the generated report recapitulated the same high-level biological interpretation as the prior expert-guided analysis. Partial recovery was assigned when the report recovered one major component of the prior finding but omitted or weakly represented another component. This retrospective validation was intended as an external expert-curated concordance analysis. For transparency, the full generated reports used for the retrospective validation judgments were retained and are provided in Appendix C.

### 2.9. Disease-Only PubMed-RAG Baseline

To address whether the proposed framework provides value beyond the general effect of giving an LLM retrieved context, we added a disease-only PubMed-RAG baseline. This baseline was designed as a non-graph alternative to SemNet Explorer. PubMed abstracts were retrieved separately for AD, ALS, and FTD using disease-level mechanistic queries: “Alzheimer’s disease mechanism neurodegeneration”, “amyotrophic lateral sclerosis mechanism neurodegeneration”, and “frontotemporal dementia mechanism neurodegeneration”. The baseline did not receive SemNet Venn-region labels, enriched process labels, anchor rankings, mediator paths, or pairwise/combined disease queries.

Using only the retrieved disease-specific abstracts, the same report-generation LLM was asked to produce a comparative report describing shared mechanisms and disease-emphasized mechanisms across AD, ALS, and FTD. We compared this disease-only PubMed-RAG baseline with SemNet KG-grounded reporting in the AAPP molecular layer, where SemNet anchors correspond most directly to mechanistic molecular entities such as receptors, peptides, proteins, and enzymes. The SemNet report used the graph-derived *R*_123_, *R*_1_, *R*_2_, *R*_3_ region structure, enriched processes, and representative anchors. An independent judge model evaluated both reports over ten repeated runs using five dimensions: biomedical plausibility, evidence faithfulness, shared/specific distinction, mechanistic specificity, and comparative clarity.

### 2.10. Application-Layer Deployment, Exported Artifacts, and Reproducibility

**Figure 3.**
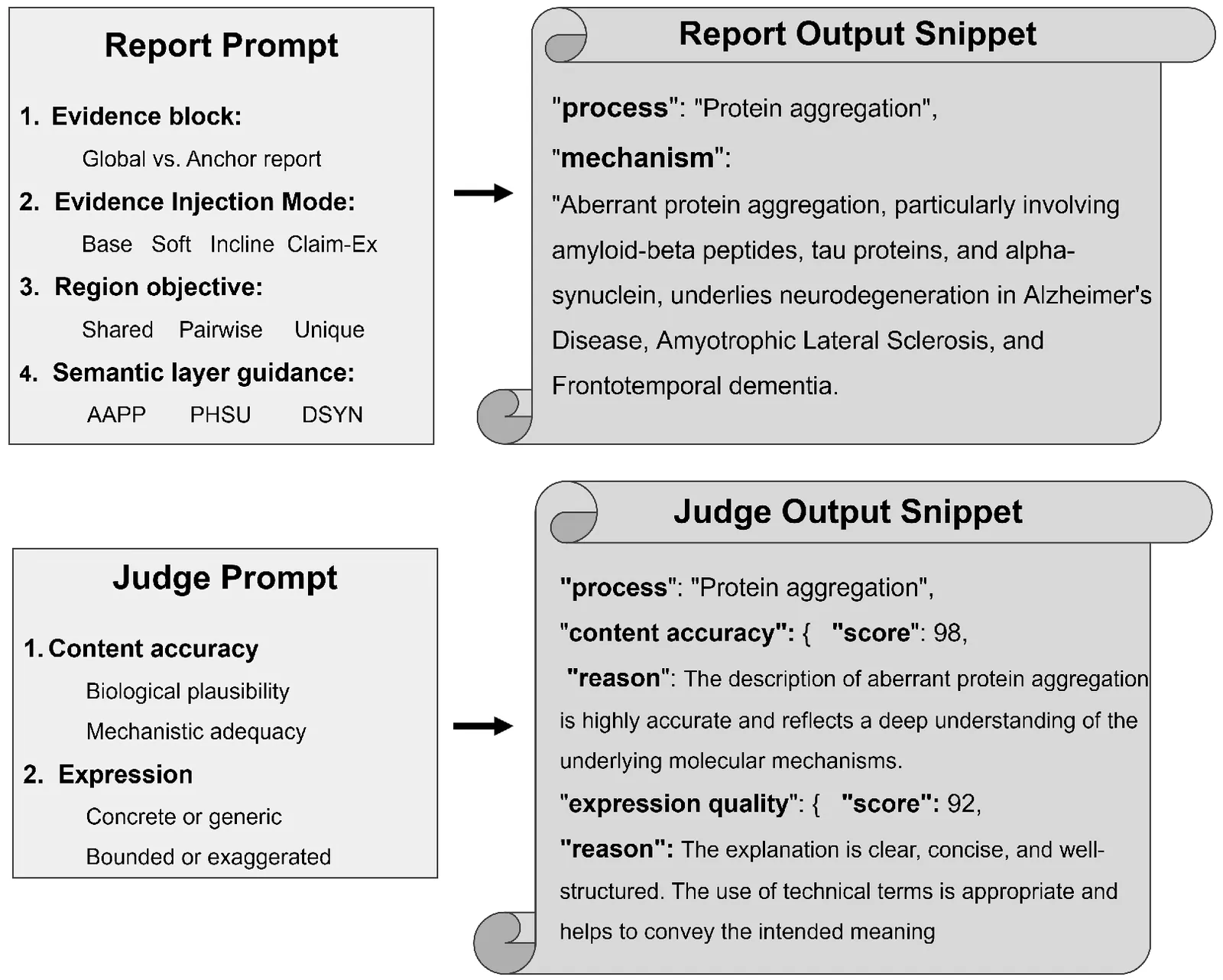
Application-layer prompt–report–judge workflow with representative output snippets. The left panels summarize the report prompt structure and judge rubric, including evidence blocks, evidence-injection modes, region objectives, semantic-layer guidance, and evaluation dimensions. The right panels show example report and judge outputs exported from the deployed system.

Figure 3 summarizes the prompt–report–judge workflow together with the policy-evaluation signals used to support application-layer deployment. The deployed application uses the ablation-derived adaptive prompt policy rather than a single fixed prompt rule. For each reporting request, the system selects the prompt mode according to report family, semantic layer, region type, and local evidence structure, and then generates the corresponding JSON report. An optional judge layer can also be invoked in the interface to review the generated output under the same two dimensions used in evaluation: **content accuracy** and **expression quality**.

To expose representative generated explanations from the deployed system, we compiled an application-layer case study directly from exported interface artifacts. We sampled representative settings covering both report families (**global** and **anchor**), two regions (shared core *R*_123_ and unique *R*_1_), and three semantic layers (AAPP, DSYN, PHSU) for the AD–ALS–FTD triad. Each sampled case was stored as a directory containing four text artifacts: report_prompt.txt, report.txt, judge_prompt.txt, and judge.txt. These exports preserve the exact prompt, response, and audit chain used by the application and allow qualitative inspection of representative generated mechanisms.

The backend loads a fixed SemNet 2.0 snapshot at server startup. Ranking outputs (CSV) and classification/enrichment outputs (region JSON) are cached and reused, ensuring deterministic candidate sets for a given graph snapshot, disease triad, and semantic layer. Prompt templates, evidence blocks, generated reports, and exported audit artifacts can be logged for transparency. Remaining nondeterminism arises primarily from LLM sampling and is partially mitigated by conservative decoding, schema validation, and optional judge auditing. This design ensures that each generated report can be traced back to its underlying evidence inputs and prompt configuration, enabling transparent inspection of the full evidence-to-report pipeline.

## 3. Results and Discussion

**Figure 4.**
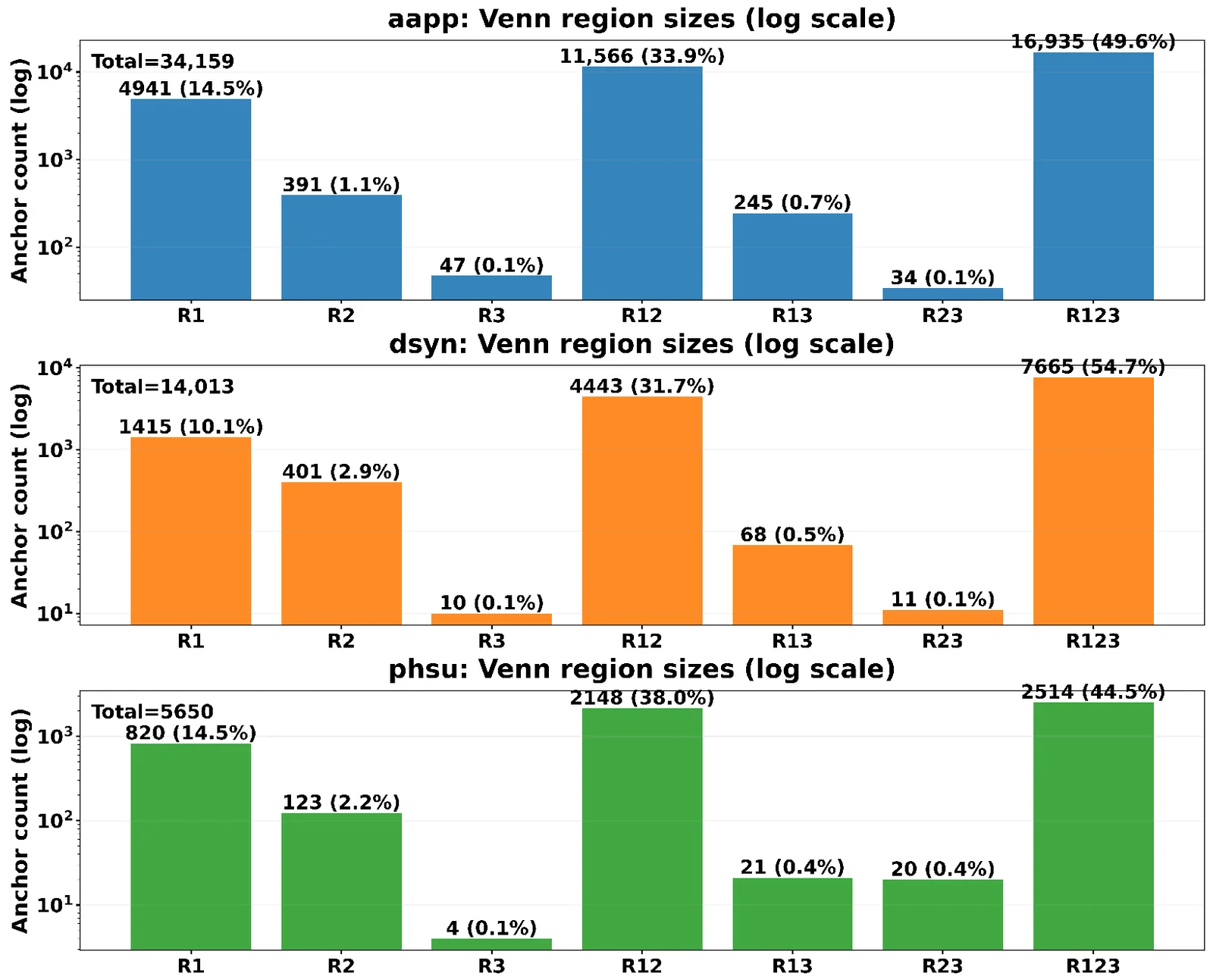
Sizes of the seven Venn regions for three semantic layers: **AAPP** (amino acids, peptides, and proteins), **DSYN** (diseases and syndromes), and **PHSU** (pharmacologic substances). The seven regions are defined as follows: *R*_123_, anchors connected to all three diseases (AD, ALS, and FTD); *R*_12_, *R*_13_, and *R*_23_, anchors connected to exactly the AD–ALS, AD–FTD, and ALS–FTD pairs, respectively; and *R*_1_, *R*_2_, and *R*_3_, anchors connected only to AD, only to ALS, and only to FTD, respectively. Bars show anchor counts on a log scale, with percentages relative to the total number of anchors in the corresponding semantic layer. In all three layers, the anchor distribution is concentrated in the shared tri-disease core *R*_123_ and the AD–ALS pairwise region *R*_12_, while the remaining pairwise regions and the FTD-only region *R*_3_ contain comparatively few anchors.

**Figure 5.**
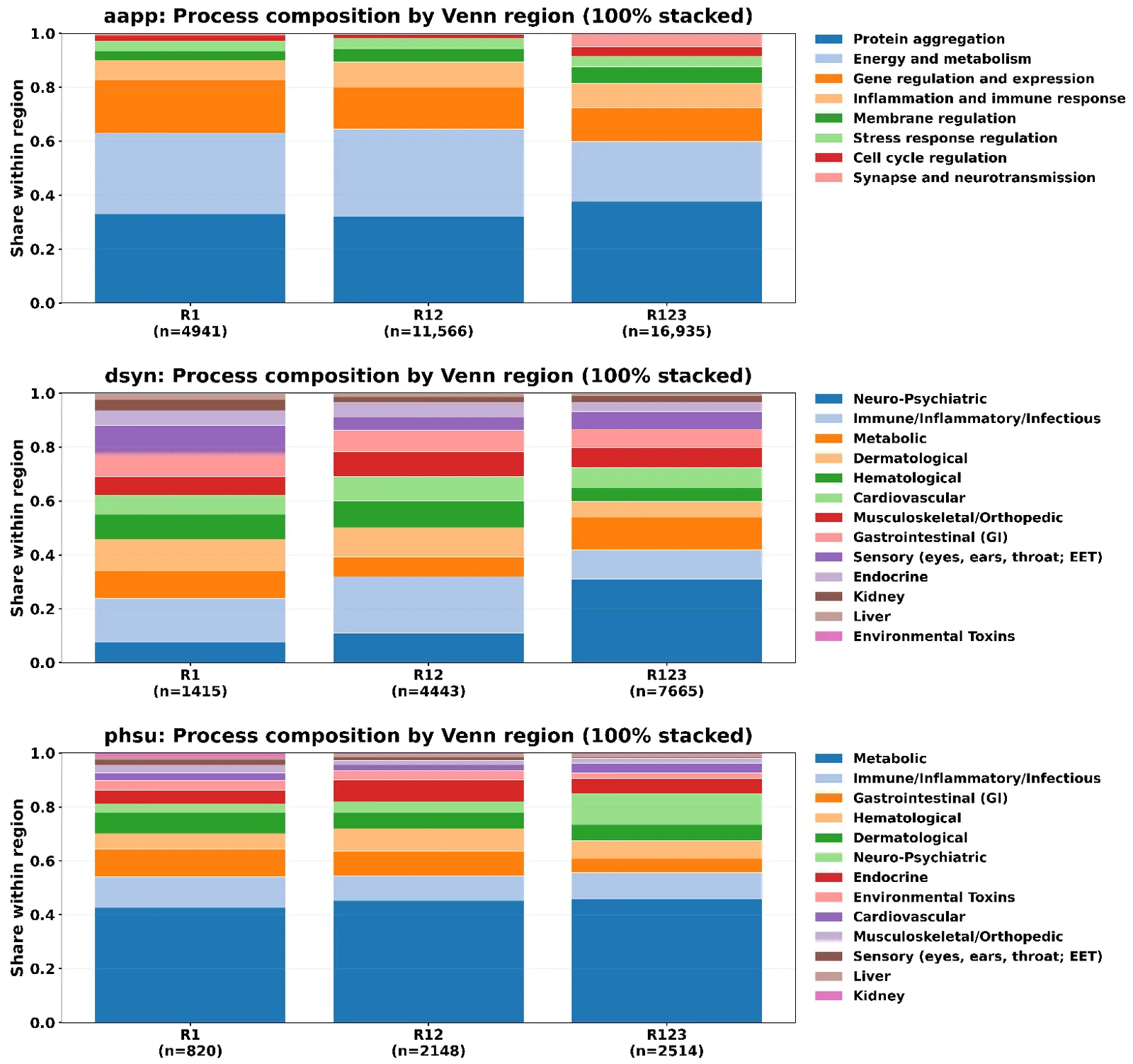
Within-region process/category composition for three selected Venn regions in three semantic layers: **AAPP** (amino acids, peptides, and proteins), **DSYN** (diseases and syndromes), and **PHSU** (pharmacologic substances). The three regions shown are *R*_123_, anchors connected to all three diseases (AD, ALS, and FTD); *R*_12_, anchors connected to the AD–ALS pair but not FTD; and *R*_1_, anchors connected only to AD. After closed-set anchor-to-process/category mapping, each stacked bar shows the relative proportion of anchors assigned to each process/category within that region, so that bar heights sum to 100%. Region sizes (*n*) are shown below each bar to indicate the number of anchors contributing to the corresponding composition profile. This view highlights how the dominant biological/process makeup differs among the shared core, the dominant pairwise overlap, and the AD-specific region across semantic layers.

**Figure 6.**
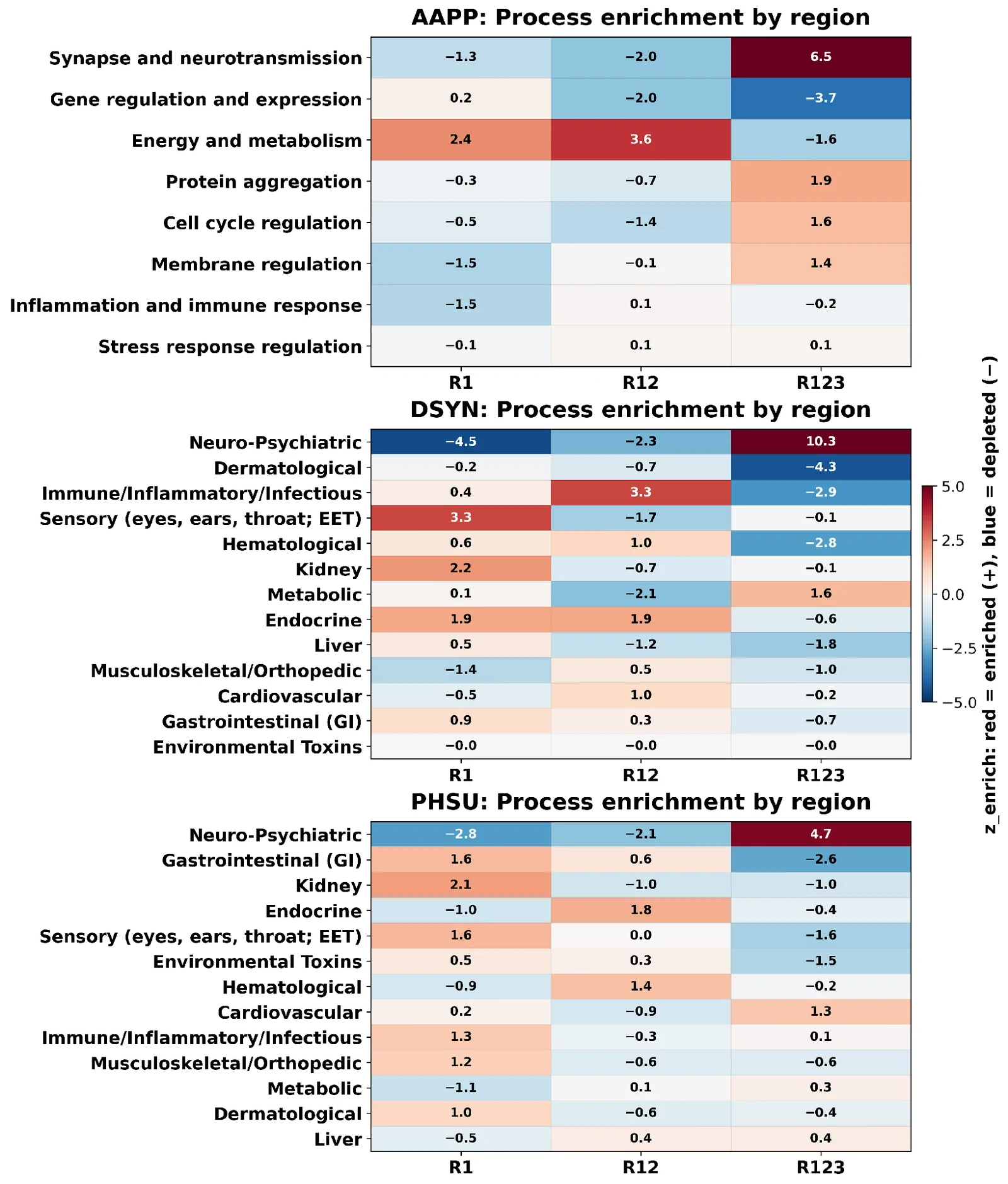
Region-level process/category enrichment heatmaps for three selected Venn regions in three semantic layers: **AAPP** (amino acids, peptides, and proteins), **DSYN** (diseases and syndromes), and **PHSU** (pharmacologic substances). The regions shown are *R*_123_, anchors connected to all three diseases (AD, ALS, and FTD); *R*_12_, anchors connected to AD and ALS but not FTD; and *R*_1_, anchors connected only to AD. Rows correspond to predefined process/category labels, and columns correspond to regions. Each cell shows the region-level enrichment score *z*_enrich_, which compares the observed number of anchors assigned to that process/category in the selected region against the expected number under the semantic-layer-specific background. Red indicates enrichment, blue indicates depletion, and the printed values show the exact *z*_enrich_ scores. This view highlights which processes/categories are specifically over- or under-represented in the shared core, the dominant pairwise overlap, and the AD-specific region.

**Figure 7.**
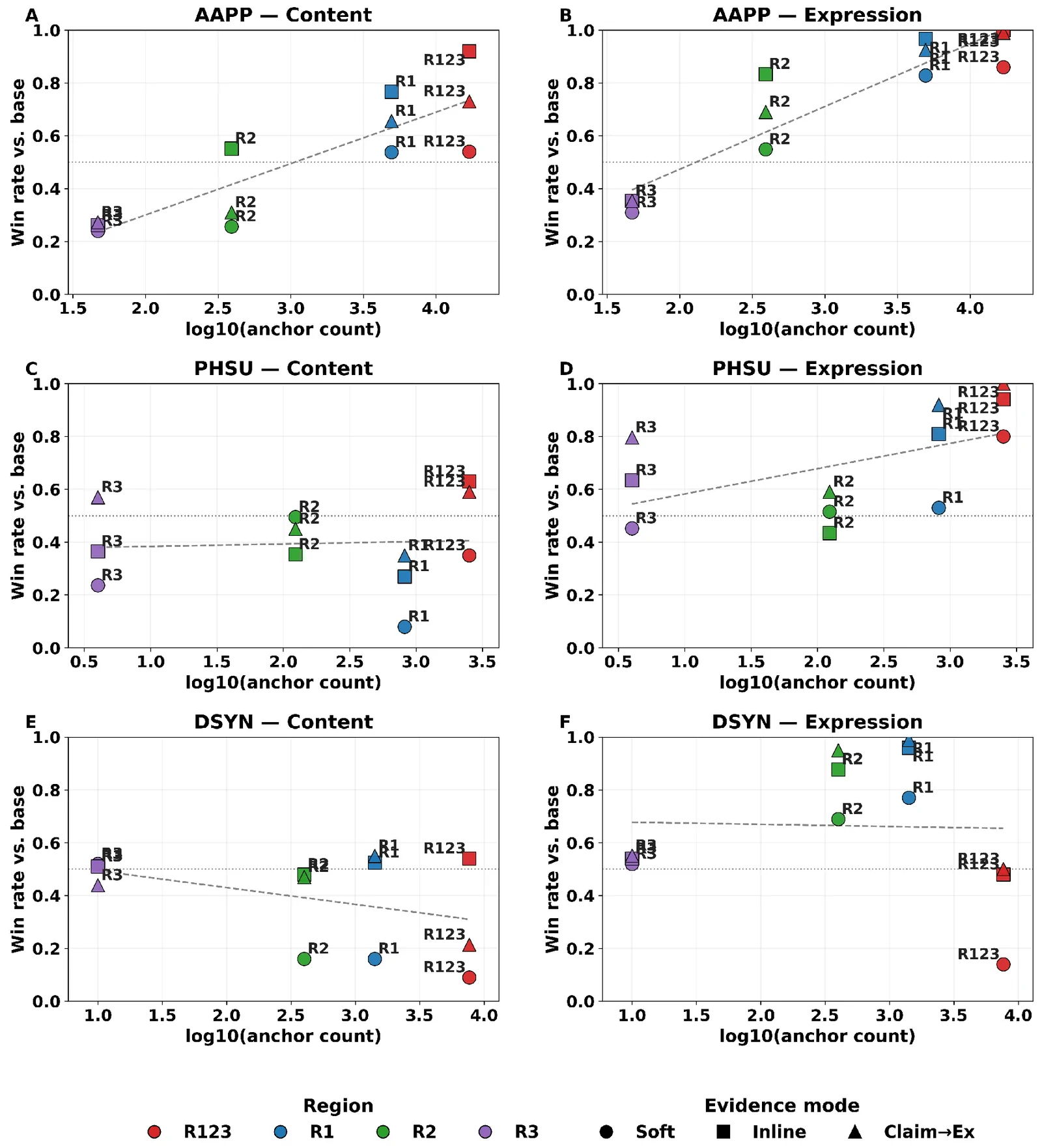
Process-level evidence-grounding ablation for global region reports across three semantic layers: **AAPP** (panels **A**,**B**), **PHSU** (panels **C**,**D**), and **DSYN** (panels **E**,**F**). The left column shows judge win rates for **content accuracy**, and the right column shows judge win rates for **expression quality**, each relative to the clean base prompt. The x-axis gives the log-transformed anchor count of the corresponding region, point color denotes region (*R*_123_, *R*_1_, *R*_2_, *R*_3_), and marker shape denotes evidence-injection mode: **soft**, **inline**, and **claim-then-example**. The horizontal dotted line at 0.5 marks parity with the base prompt, and the dashed line shows the fitted trend between evidence volume and win rate. Across layers, evidence grounding improves expression quality more consistently than content accuracy, with the strongest gains observed in larger, evidence-rich regions and lower-abstraction settings (AAPP and PHSU).

**Figure 8.**
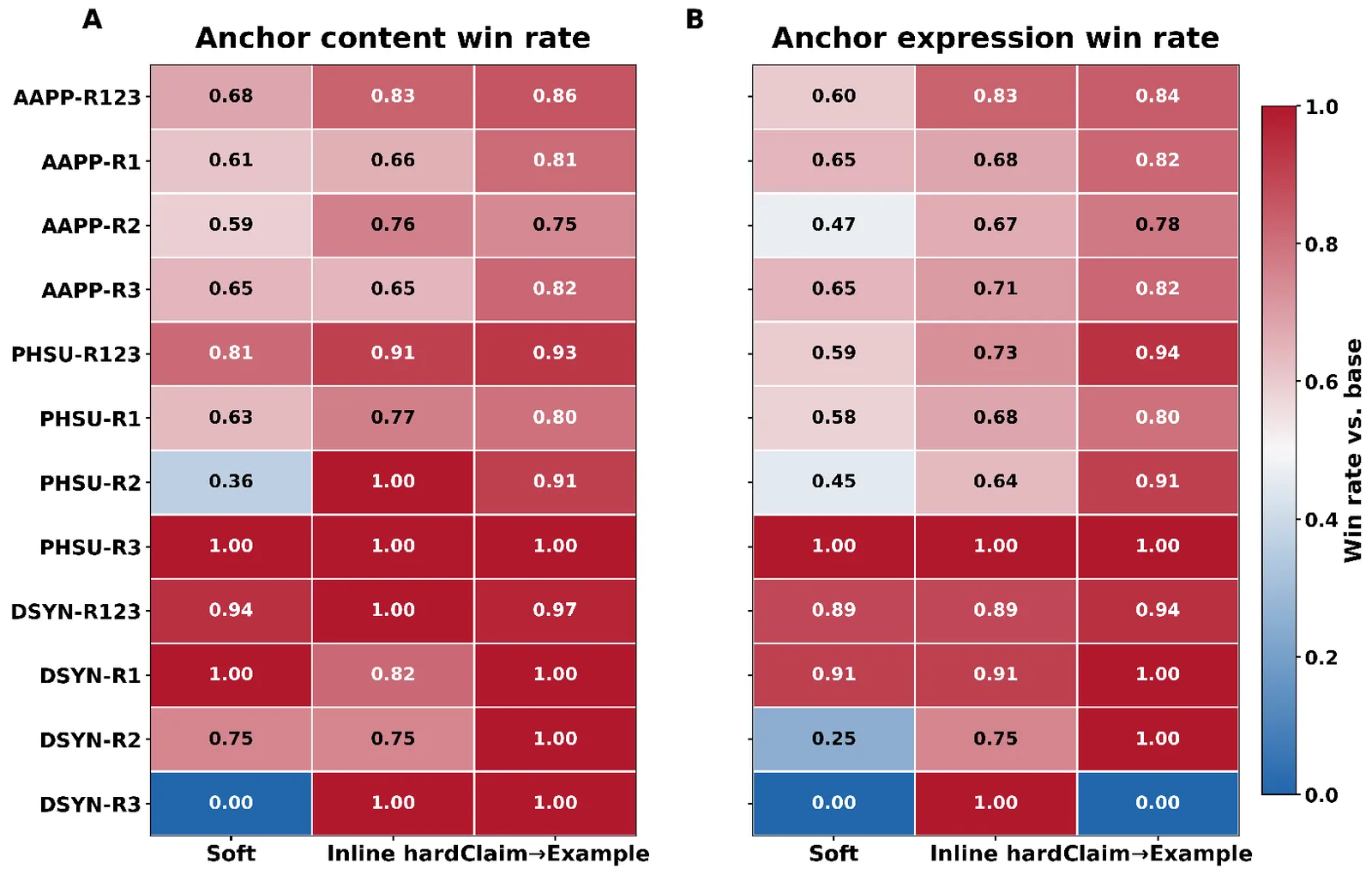
Anchor-level evidence-grounding ablation across three semantic layers and four regions. Rows correspond to **AAPP**, **PHSU**, and **DSYN**; columns correspond to judge win rates for **content accuracy** (**A**) and **expression quality** (**B**), each relative to the clean base prompt. Within each heatmap cell, the compared evidence-injection modes are **soft**, **inline-hard**, and **claim-then-example**. Warmer colors indicate stronger preference over the base prompt. In contrast to global reports, stronger mediator-constrained prompting improves both content and expression more consistently, particularly in AAPP and PHSU settings, reflecting tighter alignment between local evidence and reporting objectives.

**Figure 9.**
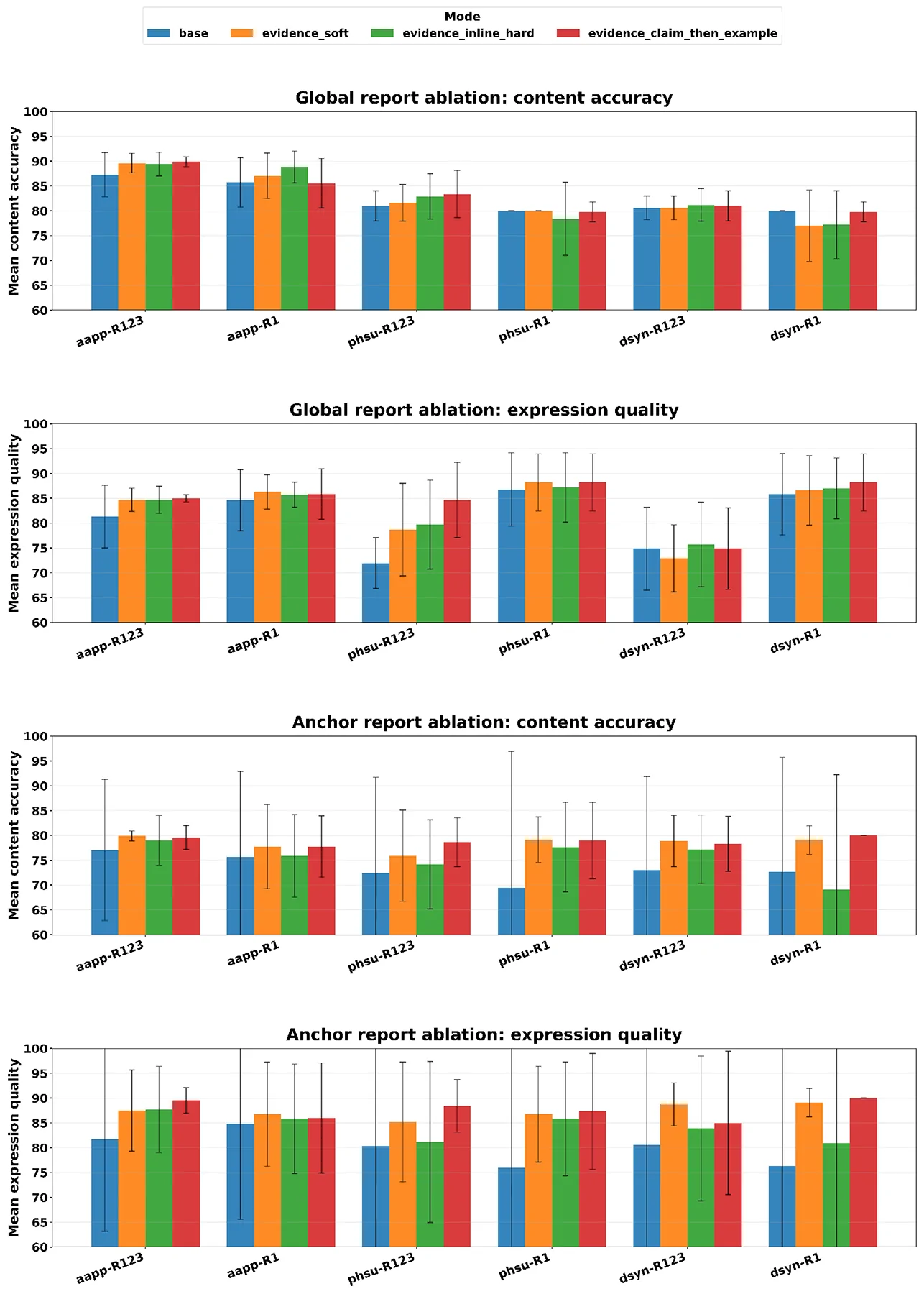
Absolute score summaries for the four prompt modes across all 12 node-type/region settings in global and anchor reporting. From top to bottom, the four panels show global-report **content accuracy**, global-report **expression quality**, anchor-report **content accuracy**, and anchor-report **expression quality**. Bars show mean judge scores and error bars show standard deviations over repeated generations. These results complement pairwise ablations by showing the overall score landscape, confirming that global reports benefit selectively from evidence grounding, whereas anchor-centric reports show more consistent improvements under stronger grounding strategies.

We applied the full workflow to a neurodegenerative disease triad consisting of *Alzheimer’s Disease* (C0002395), *Amyotrophic Lateral Sclerosis* (C0002736), and *Frontotemporal Dementia* (C0338451). To test stability across semantic abstraction levels, we ran the same pipeline on three node types: **AAPP** (amino acids, peptides, and proteins), **DSYN** (diseases and syndromes), and **PHSU** (pharmacologic substances). Figures 4-9 summarize the resulting region structures, process/category compositions, enrichment patterns, and evidence-grounding ablations. A concise summary of key takeaways is provided in Table 1.

To make Section 3 easier to follow, we organize the findings around four practical questions. First, we ask how graph-derived anchors are distributed across shared, pairwise, and disease-specific regions. Second, we ask whether process/category enrichment converts ranked anchors into interpretable report scaffolds. Third, we evaluate when explicit evidence grounding improves global and anchor-centric report generation. Fourth, we compare KG-grounded reporting with non-graph and application-layer outputs to clarify the practical value of region-aware reporting. This structure separates structural findings, reporting-quality findings, and application-level implications.

### 3.1. Region Structure Concentrates in the Shared Core and the AD–ALS Pairwise Overlap

Figure 4 shows a stable and highly uneven region distribution across all three semantic layers. In each layer, the largest compartment is the shared tri-disease core *R*_123_, followed by the AD–ALS pairwise overlap *R*_12_. For AAPP, *R*_123_ and *R*_12_ together account for 83.5% of all anchors; for DSYN, 86.4%; and for PHSU, 82.5%. In contrast, *R*_13_, *R*_23_, and *R*_3_ remain consistently small, while *R*_1_ is the largest unique region in all three layers.

This distribution matters methodologically as well as biologically. It shows that the strongest comparative signal is concentrated in the shared core and one dominant pairwise overlap, whereas other regions represent lower-support but still potentially meaningful divergence structure. In practical terms, the seven-region decomposition provides a more useful scaffold for reporting than a single pooled ranked list: *R*_123_ supports comorbidity-oriented synthesis, whereas pairwise and unique regions preserve disease-biased contrast.

### 3.2. Composition and Enrichment Convert Ranked Anchors into Interpretable Report Scaffolds

Figure 5 shows that within-region composition yields compact and interpretable summaries of regional structure. In the AAPP layer, the dominant regions are composed mainly of *Protein aggregation*, *Energy and metabolism*, and *Gene regulation and expression*, with additional contributions from *Inflammation and immune response* and *Membrane regulation*. In DSYN, the dominant regions are organized mainly around *Neuro-Psychiatric*, *Metabolic*, and *Immune/Inflammatory/Infectious* categories. In PHSU, *Metabolic* and *Neuro-Psychiatric* categories dominate, with smaller contributions from *Immune/Inflammatory/Infectious*, *Gastrointestinal*, *Endocrine*, and *Hematological*. Across layers, the major regions can therefore be summarized by compact mixtures of processes/categories rather than by long unstructured lists of anchors.

Figure 6 sharpens this picture by showing which processes are specifically over- or under-represented relative to the layer-specific background. In AAPP, the shared core *R*_123_ is most strongly enriched for *Synapse and neurotransmission* (*z* = 6.5), together with positive enrichment for *Protein aggregation*, *Cell cycle regulation*, and *Membrane regulation*, whereas *R*_12_ is enriched for *Energy and metabolism*. In DSYN, *R*_123_ is strongly enriched for *Neuro-Psychiatric* (*z* = 10.3), while *R*_12_ shifts toward *Immune/Inflammatory/Infectious*. In PHSU, *R*_123_ is again enriched for *Neuro-Psychiatric*, whereas *R*_12_ is depleted for that same category. These patterns show that *R*_123_ and *R*_12_ are both large but not redundant. Composition identifies the main axes present within a region, whereas enrichment identifies which of those axes are specifically characteristic of that region. Together, these views provide the deterministic scaffold for downstream reporting. Because *R*_12_ is a large and biologically informative compartment, it is included in the region-size, composition, and enrichment analyses. However, the subsequent prompt-ablation analysis focuses on *R*_123_ and *R*_1_, *R*_2_, *R*_3_, which correspond to the two main report objectives evaluated in this study: shared-core reporting and disease-specific divergence reporting. Thus, the pairwise regions should be interpreted here as structurally characterized but not equally validated at the report-ablation level.

### 3.3. Global Process Reports Benefit from Evidence Grounding, but Mainly in Expression and Only Conditionally in Content

Figure 7 summarizes the process-level ablation results for **global region reports**. The six panels are organized by semantic layer and evaluation dimension: panels A–B correspond to AAPP, C–D to PHSU, and E–F to DSYN; the left column shows **content accuracy** and the right column shows **expression quality**. Within each panel, point color denotes region (*R*_123_, *R*_1_, *R*_2_, *R*_3_), marker shape denotes evidence-grounding mode, and the horizontal dotted line at 0.5 marks parity with the clean base prompt. Points above 0.5 indicate that the evidence-grounded variant was preferred over the base prompt more often than not, whereas points below 0.5 indicate the opposite. The x-axis shows the log-transformed anchor count of the corresponding region, and the dashed regression line summarizes the overall relationship between evidence volume and win rate.

The clearest overall pattern is that evidence grounding improves **expression quality** more reliably than **content accuracy**. This is most visible in the right-column panels (B, D, F), where many settings exceed the 0.5 baseline, especially in larger regions. By contrast, the left-column panels (A, C, E) remain more heterogeneous, with several settings near or below parity. Thus, explicit graph-derived evidence more consistently improves clarity and supportiveness of the reports than judged biological adequacy.

The AAPP panels (A, B) show the most favorable response to evidence grounding. In panel A, the larger regions—especially *R*_123_ and *R*_1_, which appear at higher anchor counts on the right side of the plot—often exceed 0.5 for content, although not uniformly across all evidence modes. In panel B, the same regions are consistently well above 0.5 for expression, with several settings approaching 0.9 or higher. Thus, in the molecular layer, explicit anchor evidence is often beneficial for both content and expression, particularly when regional evidence is abundant.

The PHSU panels (C, D) show a more asymmetric pattern. In panel C, content gains are modest and inconsistent: some settings remain near parity, and others fall clearly below it, especially for *R*_1_. In panel D, however, expression improves much more consistently, particularly for the larger shared and pairwise regions. Thus, in the pharmacologic layer, explicit evidence improves report articulation more consistently than judged biological adequacy.

The DSYN panels (E, F) are the least stable. In panel E, most content results cluster near or below 0.5, and the high-count *R*_123_ settings are often unfavorable relative to the base prompt. In panel F, expression remains mixed: *R*_1_ and *R*_2_ show some benefit, but the *R*_123_ settings again sit around or below parity. This suggests that hard grounding becomes less reliable in the disease/syndrome layer because the evidence units are more abstract and less directly mechanistic than in AAPP.

Two broader design implications follow from Figure 7. First, the usefulness of explicit evidence depends on **evidence sufficiency**: the strongest gains are concentrated in larger, better-supported regions, especially *R*_123_ and *R*_1_, whereas sparse regions often show limited or inconsistent content improvement. Second, the effect depends on **semantic abstraction**: AAPP is the most favorable setting, PHSU is intermediate, and DSYN is the least stable. This ordering is consistent with the increasing abstraction of node types, where AAPP anchors remain closer to direct mechanistic interpretation and DSYN nodes are more disease-like and indirect.

Taken together, the global-report setting supports **adaptive evidence grounding** rather than a single fixed prompt rule. Evidence injection is often beneficial, but its effectiveness depends on both evidence sufficiency and semantic abstraction. In higher-level or evidence-aggregated settings, explicit grounding appears to improve interpretability and clarity more consistently than underlying mechanistic correctness, highlighting a distinction between evidence-supported articulation and evidence-limited inference in LLM-based scientific reporting.

### 3.4. Anchor-Centric Reports Respond More Favorably to Strong Local Grounding, and Absolute Scores Support the Same Deployment Logic

Figure 8 extends the ablation analysis from global process reports to local anchor-centric reports. Unlike Figure 7, where grounding improved mainly **expression quality**, Figure 8 shows a more consistently favorable pattern across both **content accuracy** and **expression quality**. In the heatmaps, warmer cells indicate that an evidence-grounded mode defeats the clean base prompt more often than not. Across most node-type/region combinations, particularly in AAPP and PHSU, stronger grounding modes outperform the base prompt in both dimensions.

This difference is most apparent in the overall heatmap structure. In AAPP, both content and expression frequently improve under stronger grounding, particularly in the shared core *R*_123_ and larger disease-specific regions. PHSU shows a similar but slightly weaker trend, whereas DSYN remains less stable, especially in unique regions. Even so, the anchor-level setting remains more favorable to grounding than the corresponding global-report setting. The main reason is that anchor reports are narrower tasks: the model explains one anchor using a small local mediator set rather than synthesizing several process axes across an entire region. Because mediator evidence is more directly aligned with the reporting target, stronger grounding improves not only report wording, but also judged mechanistic adequacy.

The heatmaps also show a clear ordering among evidence-injection modes. Across many settings, **claim-then-example** is the warmest or tied-warmest cell, with **inline-hard** usually the next most favorable mode. By contrast, **soft** evidence use is less consistently dominant. This indicates that anchor-centric reporting benefits from a structured local pattern in which the model first states the main mechanistic interpretation and then supports it with mediator evidence, rather than merely being given evidence as optional context. Although a few DSYN unique-region settings remain unstable, the overall pattern is substantially more favorable to strong grounding than in the global-report setting.

Figure 9 complements the pairwise win-rate analysis by showing the *absolute* score profile of each mode across all 12 node-type/region settings. The top two panels correspond to global reports and the bottom two to anchor reports; within each pair, the first panel shows **content accuracy** and the second shows **expression quality**. Unlike the pairwise ablations, which measure relative preference against the base prompt, these panels show the overall score landscape across the full deployment matrix.

For **global reports**, the absolute-score panels reinforce the same conclusion drawn from Figure 7. Evidence grounding often improves **expression quality**, especially in the larger AAPP and PHSU settings, but **content accuracy** remains heterogeneous. In several sparse or more abstract settings, particularly in DSYN, the base prompt remains competitive or even preferable. Thus, the absolute scores reinforce that global reporting requires setting-dependent grounding rather than one rigid evidence template.

For **anchor reports**, the bottom two panels show a much more favorable score landscape for strong grounding. Relative to the base prompt, the stronger evidence-injection modes improve both **content accuracy** and **expression quality** more broadly. Again, **claim-then-example** is frequently the strongest or tied-strongest mode, with **inline-hard** serving as the most reliable secondary strategy. This is consistent with Figure 8, where the same two modes most often outperform the base prompt in pairwise comparison.

Figures 8 and 9 provide two complementary views of the same deployment logic. The pairwise heatmaps show which modes are preferred over the baseline under matched conditions, whereas the bar plots show how those same modes behave in absolute score terms across the full matrix of node types and regions. Together, these views strengthen the main design conclusion of this study: This contrast indicates that the effectiveness of grounding depends on alignment between evidence granularity and reporting objective, with localized, mechanism-specific contexts benefiting more directly from strong evidence constraints.

### 3.5. Post Hoc Statistical Interpretation and Failure Analysis

We further performed a post hoc statistical analysis of the repeated judge comparisons to distinguish robust improvements from directional trends. In the global *R*_123_ reports, AAPP showed robust improvement for inline-hard evidence in both content accuracy and expression quality. For content accuracy, inline-hard evidence achieved a win rate of 0.92, Wilson 95% CI of 0.850–0.959, and exact binomial *p* = 1.91 × 10^−15^. PHSU also showed robust improvement for inline-hard evidence in content accuracy, with a win rate of 0.63, Wilson 95% CI of 0.532–0.718, and *p* = 0.012. In contrast, DSYN showed only directional improvement for inline-hard content accuracy, with a win rate of 0.54, Wilson 95% CI of 0.443–0.634, and *p* = 0.484. These results indicate that the adaptive policy reflects both statistically robust gains and weaker empirical trends, rather than uniformly definitive improvements across all semantic layers. (See Table 2).

The same analysis also clarified where evidence grounding becomes sparse or unstable. In anchor-centric reporting, AAPP-*R*_1_ had high evidence availability, with 85 successful anchors out of 100 processed anchors. By contrast, DSYN-*R*_2_ had only 4 successful anchors out of 100, and PHSU-*R*_2_ had 11 successful anchors out of 100. The main failure mode was not malformed JSON or judge failure, but empty mediator evidence for a selected anchor-region pair. Therefore, high win rates in sparse settings such as DSYN-*R*_3_ or PHSU-*R*_3_, where only one anchor was successfully evaluated, were treated as descriptive rather than statistically robust.

### 3.6. AAPP Molecular-Layer Comparison with Disease-Only PubMed-RAG

To further isolate the contribution of graph-derived structure, we compared SemNet Explorer with a disease-only PubMed-RAG baseline in the AAPP molecular layer. This comparison was not intended to test whether KG-grounded reporting universally dominates literature RAG. Instead, it tested whether SemNet-derived region structure improves contrastive molecular reporting when the task requires distinguishing shared-core and disease-emphasized mechanisms. (See Table 3).

Across ten repeated comparisons, the independent judge preferred the SemNet KG-grounded AAPP report over the PubMed-RAG report in all overall comparisons (10/10). The largest differences were observed for shared/specific distinction, mechanistic specificity, and comparative clarity. PubMed-RAG generated plausible broad neurodegeneration summaries, but it tended to rely on general disease-level mechanisms inferred from the retrieved abstracts. In contrast, the SemNet KG-grounded report explicitly preserved the graph-derived *R*_123_, *R*_1_, *R*_2_, *R*_3_ structure and used enriched AAPP processes and representative anchors to organize shared and disease-emphasized molecular mechanisms.

These results clarify the specific gain of the KG-grounded design. The PubMed-RAG baseline had access to disease-specific textual evidence and therefore produced biologically plausible summaries. However, because it did not receive graph-derived region membership, it had to infer similarity and difference indirectly from a limited disease-level retrieval sample. SemNet Explorer instead organized evidence before generation through Venn-region membership, process enrichment, and anchor ranking. The improvement in shared/specific distinction and mechanistic specificity therefore supports the claim that the framework contributes structured, region-aware evidence organization, rather than merely adding more context to the LLM.

### 3.7. Final Adaptive Prompt Policy Used in Deployment

Because the adaptive policy is used by the deployed application, we state the final prompt-selection rules explicitly in Table 4. The policy is report-family specific. For global process reports, the selected mode reflects the evidence-injection strategy with the highest content-accuracy win rate, with fallback to the base prompt when no grounded mode exceeded the 0.5 parity threshold. For anchor-centric reports, the same content-first rule was used, with expression quality as the secondary criterion. This table makes the deployment policy explicit rather than requiring readers to reconstruct it from the ablation figures.

The final policy reflects the main pattern observed in the ablation results. Global reports require more selective grounding: several settings fall back to the base prompt, especially in sparse or more abstract regions such as AAPP-*R*_3_, PHSU-*R*_1_, PHSU-*R*_2_, and DSYN-*R*_2_. In contrast, anchor-centric reports almost always select a stronger mediator-grounded mode, most often claim-then-example or inline-hard evidence. This supports the interpretation that local mediator evidence is better aligned with anchor-level reporting objectives, whereas global process reports require more cautious, setting-dependent grounding.

### 3.8. Application-Layer Examples Show That the Deployed System Produces Distinct Explanation Styles Across Report Families, Regions, and Semantic Layers

To make the deployed output concrete, we inspected representative exported cases spanning semantic layers, regions, and report families. Here we summarize the main generated content, while longer excerpts are provided in Appendix A.

Among the **global reports**, the DSYN–*R*_123_ case generated a shared *Neuro-Psychiatric* explanation centered on *Neurodegenerative Disorders* and *Cerebral atrophy*, with the report explicitly describing shared *cognitive and motor impairments* and *progressive brain atrophy and dementia* across AD, ALS, and FTD. The corresponding judge scores were 80 for content accuracy and 70 for expression quality. In the same shared-core setting, the AAPP–*R*_123_ case generated a *Protein aggregation* mechanism centered on *amyloid-beta peptides*, *tau proteins*, and *alpha-synuclein*, and described *proteostasis disruption*, *oxidative stress*, and *impaired neuronal function*; this report received **90** for content accuracy and **85** for expression quality.

The **unique-region global reports** shifted toward Alzheimer’s-biased contrastive explanations. In the AAPP–*R*_1_ case, under *Energy and metabolism*, the generated report emphasized *Apolipoprotein E (APOE)*, *amyloid-beta clearance*, *lipid transport*, *amyloid plaques*, *neuroinflammation*, *oxidative stress*, and *mitochondrial dysfunction*; the judge scores were 90 for content accuracy and 85 for expression quality. In the DSYN–*R*_1_ case, the generated *Endocrine* explanation referred to *steroid-induced diabetes*, *pituitary incidentaloma*, *chronic inflammation*, and *metabolic changes* as Alzheimer’s-biased syndrome features, with judge scores of 80 and 90.

The **anchor-centric reports** showed a more localized style. In the PHSU–*R*_123_ anchor case, the selected anchor *Acetylcholinesterase Inhibitors* was linked to *cholinergic pathways*, *Amyloid Precursor Protein (APP)*, *amyloid-beta processing*, and *glutamatergic signaling*, including mention of *memantine*; the corresponding judge scores were 80 for content accuracy and 90 for expression quality. In the DSYN–*R*_123_ anchor case, the selected anchor *Parkinson Disease* was explained through *post-translational protein processing*, *misfolded proteins*, *impaired glucose metabolism*, and *shared gene modules*, again with judge scores of 80 and 90.

The **unique-region anchor reports** again shifted toward disease-specific mechanisms. In the AAPP–*R*_1_ anchor case, the selected anchor *Cytochrome P-450 CYP2D6* was linked to *neuroprotective and neurotoxic pathways*, *amyloid-beta and tau pathologies*, *prasterone metabolism*, *neurosteroid-mediated neuroprotection*, *xenobiotic metabolism*, *oxidative stress*, and *proteotoxicity*; this report received 80 for content accuracy and 90 for expression quality.

Taken together, these cases show that the deployed system generates clearly different kinds of explanations across settings. Shared-core global reports emphasize broad cross-disease processes, unique-region global reports emphasize Alzheimer’s-biased contrast, shared-core anchor reports emphasize localized multimorbidity mechanisms, and unique-region anchor reports emphasize disease-specific local mechanisms. The semantic-layer difference is also visible in the generated wording itself: AAPP reports are more molecular, with terms such as amyloid-beta, tau, APP, oxidative stress, and proteostasis, whereas DSYN reports are broader and more syndrome-level, with terms such as cerebral atrophy, neurodegenerative disorders, endocrine dysregulation, and shared clinical burden.

### 3.9. Retrospective Validation Recovers Major Expert-Derived Findings Across Prior SemNet Studies

To address whether the generated reports recover biologically meaningful findings beyond internal prompt comparisons, we evaluated SemNet Explorer against five prior expert-curated SemNet 2.0 studies. The validation cases covered comparative pathophysiology, multimorbidity/comorbidity analysis, drug repurposing, adverse-event prediction, and mechanistic systems biology. Across these cases, SemNet Explorer generally recovered the major expert-derived themes at either the global region level, the anchor-centric level, or both (Table 5). The complete generated validation reports used to support these recovery judgments are provided in Appendix C.

For the AD–ALS–FTD comparative pathophysiology case, the shared-core AAPP report recovered synaptic/neurotransmission and protein-aggregation mechanisms across the three neurodegenerative diseases. In this run, inflammatory signaling was less explicit, so the case was interpreted as partial recovery rather than full reproduction of the prior finding. For the AD–ALS–FTD multimorbidity case, the DSYN reports recovered metabolic and neuropsychiatric disease-system organization, with pairwise regions involving FTD supporting its bridge-like role in the multimorbidity space.

For the Parkinson’s disease drug-repurposing case, the antihistamine anchor report recovered histamine-linked therapeutic rationale, including neuroinflammation, oxidative stress, dopaminergic regulation, and neurotransmitter-related mechanisms. For the CML TKI adverse-event case, the global report partially recovered shared inflammatory and metabolic adverse-event structure, while anchor-centric reports more clearly recovered drug-specific adverse-event mechanisms: ponatinib was linked to cardiovascular biology through vascular, endothelial, nitric oxide, and coagulation-related mechanisms, whereas nilotinib was linked to diabetes/metabolic biology through inflammatory cytokines, insulin resistance, and glucose metabolism. For the DKD signaling case, anchor-centric reports recovered VEGF, NF-*κ*B, MAPK/ERK, and JAK/STAT signaling modules, supporting endothelial-inflammatory and oxidative-stress mechanisms.

Overall, this retrospective analysis supports the biological concordance of SemNet Explorer reports with prior expert-guided SemNet studies. The results should not be interpreted as exact replication of prior rankings or quantitative SemNet scores. Instead, they show that the automated reporting workflow can resurface major mechanistic themes that previously required manual interpretation and domain-expert synthesis.

### 3.10. SemNet Explorer Extends Prior SemNet 2.0 Studies by Automating Report Generation and Deepening Evidence from Anchors to Mediators

Table 6 summarizes the main differences between the presented SemNet Explorer platform and the prior SemNet 2.0 studies on the AD–ALS–FTD triad. The earlier studies established that meaningful shared and disease-specific structure could be recovered from SemNet 2.0 at the AAPP, DSYN, and PHSU layers, but the final biological interpretation was still written manually at the manuscript level [11,12]. In that sense, those studies primarily stopped at graph analysis followed by manual interpretation.

The present work extends that line in two main ways. First, it moves from **manual interpretation** to **LLM-assisted automated reporting**, generating structured global and anchor-centric reports directly from graph-derived evidence under explicit evidence constraints. Second, it extends the evidence depth from **anchors** to **mediators**. Whereas prior studies mainly interpreted ranked anchors together with mapped processes or categories, the present system also uses anchor–mediator–disease structure to support localized mechanistic drill-down.

At the same time, the biological conclusions remain broadly consistent with those prior studies. The AAPP results recover similar molecular themes, including protein aggregation, synapse/neurotransmission, and metabolism, whereas the DSYN and PHSU layers remain broadly consistent with the previously reported shared metabolic and immune backbone [11,12]. Thus, the primary advance is not a change in the underlying biological interpretation, but the transformation of that interpretation into an inspectable and reusable reporting framework. This shift toward structured, automated interpretation is consistent with broader trends in AI-assisted biomedical discovery and knowledge-driven hypothesis generation [24].

A practical implication is the reduction in analysis time and effort. In prior SemNet workflows, generating a comparative mechanistic narrative could require hours to days of manual inspection and synthesis across semantic layers. In contrast, SemNet Explorer produces structured, evidence-grounded reports within seconds once inputs are specified. This enables rapid iteration across disease combinations and analysis settings, transforming comparative analysis from a labor-intensive process into a scalable interactive workflow.

The examples further illustrate that the system reproduces previously observed biological patterns in a structured, reproducible, and rapidly generated format, enabling more consistent interpretation across analysis settings.

### 3.11. Main Implications and Limitations

Several main implications follow from these results regarding how knowledge graph analyses can be transformed into scalable and reproducible scientific interpretation frameworks. First, the combination of region decomposition, process/category composition, and region-level enrichment provides a practical explanation scaffold beyond ranked lists alone by converting graph-derived overlap structure into shared, pairwise, and disease-specific report objects. Second, the ablation results show that evidence grounding should not be treated as a single fixed prompt rule. For **global reports**, its benefit is conditional and strongest in evidence-rich, lower-abstraction settings, whereas **anchor-centric reports** benefit more consistently because local mediator evidence is more tightly aligned with the reporting objective. Although demonstrated here on the AD–ALS–FTD triad, the framework is general and can be applied to a wide range of knowledge graph–based biomedical tasks, including multimorbidity analysis, comparative pathophysiology, drug target identification and repurposing, and adverse event discovery. Its ability to operate consistently across molecular (AAPP), disease-level (DSYN), and pharmacologic (PHSU) representations enables unified analysis across these domains.

In other words, the judge is most useful as a consistency-oriented evaluator of relative report quality rather than a substitute for expert biological validation.

This study also has several limitations. The SemNet Explorer platform is only as complete as the underlying SemNet 2.0 snapshot, so missing, noisy, or unevenly represented literature-derived edges may influence discovered anchors, mediator sets, and enrichment structure. The use of bounded two-hop chains is a deliberate trade-off that improves auditability, compactness, and interface usability, but it may miss longer-range mechanisms that require deeper path exploration. Finally, although classification and judging are constrained by closed-set labels and strict JSON schemas, they still depend on LLM behavior and should therefore be interpreted as structured computational assistance rather than formal reasoning.

The post hoc failure analysis also shows that strong evidence grounding depends on local evidence availability. Anchor-centric reports were most reliable when selected anchors had sufficient mediator candidates, as in AAPP-*R*_1_. In sparse settings, especially DSYN-*R*_2_, DSYN-*R*_3_, PHSU-*R*_2_, and PHSU-*R*_3_, many anchors lacked usable region-specific mediator evidence. These cases limit the statistical strength of the corresponding win-rate estimates and suggest that future versions should incorporate explicit evidence-sufficiency checks before applying strong mediator-grounded prompting.

## 4. Conclusions

We presented SemNet Explorer, an interactive evidence-grounded framework for mechanistic reporting across biomedical domains using SemNet 2.0. The SemNet Explorer platform combines bounded-hop anchor discovery, evidence-aware ranking, seven-region Venn decomposition, closed-set process classification, enrichment-derived report scaffolds, and constrained LLM report generation under explicit evidence context.

Across three semantic layers (AAPP, PHSU, and DSYN) for the AD–ALS–FTD triad, the system produced stable region structure and interpretable process-level contrasts between the tri-disease core and disease-specific regions. The ablation studies showed that effective grounding depends on explanatory granularity. At the **global process** level, explicit graph-derived evidence improves expression more consistently than content, and the benefit depends on evidence sufficiency and semantic abstraction. At the **anchor** level, stronger mediator-constrained prompting improves both judged content and expression more consistently, with claim-then-example emerging as the strongest overall strategy. Absolute score summaries support the same overall interpretation.

The application-layer case study further demonstrated that the deployed interface preserves stable prompt–report–judge workflows while adapting evidence usage and reporting structure across semantic layers and region types. More broadly, this work shifts knowledge graph analysis from a largely manual interpretive process toward an inspectable and reusable reporting framework.

The central implication is that effective integration of knowledge graphs and LLMs requires adaptive, context-dependent evidence grounding rather than fixed prompting strategies. By coupling structured graph evidence with multi-scale reporting and adaptive grounding, SemNet Explorer provides a generalizable framework for scalable scientific interpretation across biomedical domains, including multimorbidity analysis, comparative pathophysiology, drug repurposing, and adverse event discovery. More broadly, the framework demonstrates how structured graph organization can support reproducible mechanistic reporting beyond conventional retrieval-augmented generation and static literature synthesis.

## Figures and Tables

**Table 1.** Concise takeaways across semantic layers, regions, and report families.

Comparison Axis	Main Observation	Practical Implication	Most Relevant Evidence
Semantic layer	AAPP showed the clearest benefit from explicit evidence grounding; PHSU was intermediate; DSYN was less stable.	Molecular anchors are more directly mechanistic, while disease/syndrome nodes require more cautious grounding.	Figures 7-9; Section 3.5
Region type	*R*_123_ supports shared-core reporting, while *R*_1_, *R*_2_, *R*_3_ support disease-specific divergence. Pairwise regions are structurally characterized but not ablated at the same depth.	Region-aware organization helps separate comorbidity-oriented and disease-specific explanations.	Figures 4-6
Report family	Global reports benefit conditionally from grounding, mostly in expression quality; anchor-centric reports benefit more consistently from mediator-constrained grounding.	Prompt strength should depend on evidence granularity and report objective rather than using one fixed prompt.	Figures 7-9; Section 3.7
Baseline comparison	SemNet KG-grounded reporting outperformed disease-only PubMed-RAG for AAPP contrastive molecular reporting.	The main gain comes from graph-derived region organization, not simply from giving the LLM more context.	Section 3.6
Failure behavior	Sparse mediator evidence limited some anchor-centric settings, especially DSYN-*R*_2_, DSYN-*R*_3_, PHSU-*R*_2_, and PHSU-*R*_3_.	Strong grounding should be used with evidence-sufficiency checks in sparse or abstract settings.	Section 3.5; failure analysis paragraph

**Table 2.** Post hoc statistical interpretation of selected evidence-injection comparisons. Wilson 95% confidence intervals and exact binomial tests were computed against a null win probability of 0.5.

Family	Layer	Region	Mode	Dimension	Win Rate	95% CI	Interpretation
Global	AAPP	*R*_123_	inline-hard	Content	0.92	0.850–0.959	Robust
Global	AAPP	*R*_123_	inline-hard	Expression	1.00	0.963–1.000	Robust
Global	DSYN	*R*_123_	inline-hard	Content	0.54	0.443–0.634	Directional
Global	PHSU	*R*_123_	inline-hard	Content	0.63	0.532–0.718	Robust
Anchor	AAPP	*R*_1_	claim-then-example	Content	0.812	0.716–0.881	Robust
Anchor	AAPP	*R*_2_	inline-hard	Content	0.765	0.632–0.860	Robust
Anchor	PHSU	*R*_1_	claim-then-example	Content	0.800	0.682–0.882	Robust
Anchor	DSYN	*R*_2_	claim-then-example	Content	1.000	0.510–1.000	Sparse descriptive

**Table 3.** Disease-only PubMed-RAG versus SemNet KG-grounded reporting in the AAPP molecular layer. Scores are judge means and standard deviations over ten repeated comparisons.

Evaluation Dimension	PubMed-RAG	SemNet KG	KG Win Rate
Biomedical plausibility	4.0 ± 0.0	4.9 ± 0.3	0.90
Evidence faithfulness	4.3 ± 0.46	4.7 ± 0.46	0.70
Shared/specific distinction	3.4 ± 0.49	5.0 ± 0.0	1.00
Mechanistic specificity	3.0 ± 0.0	5.0 ± 0.0	1.00
Comparative clarity	3.7 ± 0.46	5.0 ± 0.0	1.00
Overall preference	–	–	1.00

**Table 4.** Final adaptive prompt policy used in deployment. The selected mode is shown for each report family, semantic layer, and region. Scores denote the content-accuracy win rate used for policy selection. “Base” indicates fallback when no evidence-grounded mode exceeded the 0.5 parity threshold.

Report Family	Layer	Region	Selected Mode	Content Win Rate	Fallback to Base
Global	AAPP	*R*_123_	inline-hard evidence	0.920	No
Global	AAPP	*R*_1_	inline-hard evidence	0.767	No
Global	AAPP	*R*_2_	inline-hard evidence	0.551	No
Global	AAPP	*R*_3_	base	0.273	Yes
Global	PHSU	*R*_123_	inline-hard evidence	0.630	No
Global	PHSU	*R*_1_	base	0.350	Yes
Global	PHSU	*R*_2_	base	0.495	Yes
Global	PHSU	*R*_3_	claim-then-example	0.570	No
Global	DSYN	*R*_123_	inline-hard evidence	0.540	No
Global	DSYN	*R*_1_	claim-then-example	0.550	No
Global	DSYN	*R*_2_	base	0.480	Yes
Global	DSYN	*R*_3_	soft evidence	0.520	No
Anchor	AAPP	*R*_123_	claim-then-example	0.860	No
Anchor	AAPP	*R*_1_	claim-then-example	0.812	No
Anchor	AAPP	*R*_2_	inline-hard evidence	0.765	No
Anchor	AAPP	*R*_3_	claim-then-example	0.824	No
Anchor	PHSU	*R*_123_	claim-then-example	0.927	No
Anchor	PHSU	*R*_1_	claim-then-example	0.800	No
Anchor	PHSU	*R*_2_	inline-hard evidence	1.000	No
Anchor	PHSU	*R*_3_	soft evidence	1.000	No
Anchor	DSYN	*R*_123_	inline-hard evidence	1.000	No
Anchor	DSYN	*R*_1_	claim-then-example	1.000	No
Anchor	DSYN	*R*_2_	claim-then-example	1.000	No
Anchor	DSYN	*R*_3_	inline-hard evidence	1.000	No

**Table 5.** Retrospective validation against prior expert-curated SemNet studies. Recovery was assessed qualitatively at the level of major mechanistic themes.

Prior Study	Task Type	Expert-Derived Finding	SemNet Explorer Recovery	Outcome
AD–ALS–FTD molecular overlap [11]	Comparative pathophysiology	Shared inflammatory and synaptic mechanisms across neurodegenerative diseases.	Recovered shared synaptic/neurotransmission and protein-aggregation mechanisms; inflammatory signaling was less explicit.	Partial recovery
AD–ALS–FTD multimorbidity [12]	Multimorbidity/comorbidity	Shared metabolic–immune multimorbidity core and FTD bridge-like organization.	Recovered metabolic and neuropsychiatric disease-system organization, with FTD-associated pairwise regions supporting bridge-like structure.	Partial recovery
Parkinson"s disease antihistamine repurposing [22]	Drug repurposing	Antihistamines identified as promising adjuvant therapies with oxidative-stress, inflammatory, and neurotransmitter mechanisms.	Recovered antihistamine-related rationale through histamine receptor modulation, neuroinflammation, oxidative stress, dopaminergic regulation, and neurotransmitter mechanisms.	Recovered at anchor level
CML TKI adverse events [21]	Adverse-event prediction	TKI therapy associated with cardiometabolic, inflammatory, renal, and hematologic adverse-event clusters; drug-specific patterns including ponatinib–cardiovascular and nilotinib–diabetes.	Partially recovered shared inflammatory and metabolic adverse-event structure globally; anchor reports recovered ponatinib-associated cardiovascular biology and nilotinib-associated diabetes/metabolic biology.	Partial global recovery; recovered at anchor level
Diabetic kidney disease signaling [19]	Mechanistic systems biology	Endothelial-inflammatory signaling dysregulation involving VEGF, NF-*κ*B, ERK/MAPK, JAK/STAT, oxidative stress, and macrophage/cytokine signaling.	Recovered VEGF, NF-kB, mapk/erk, and JAK/STAT anchor-level signaling modules supporting endothelial-inflammatory and oxidative-stress mechanisms.	Recovered at anchor level

**Table 6.** Main differences between prior SemNet 2.0 studies on the AD–ALS–FTD triad and the present unified framework.

Aspect	Prior SemNet 2.0 Studies	Present Platform
Application scope	Disease-specific comparative analyses	Multi-domain framework supporting disease comparison, drug repurposing, and adverse event analysis.
Report generation	Biological interpretation was written manually at the manuscript level after graph analysis.	Mechanistic reports are generated automatically with LLMs under explicit evidence constraints.
Evidence depth	Interpretation mainly relied on ranked anchors/nodes and their process or category mappings.	Reporting uses both anchor-level evidence and mediator-level drill-down evidence for local explanation.
Output form	Static manuscript figures and narrative discussion.	Structured JSON reports, exported artifacts, and interactive interface output.
Workflow	Fixed comparative analyses for a predefined study setting.	Reusable reporting framework that routes evidence by Venn region and report type.
Role of LLMs	LLMs mainly supported mapping, labeling, or validation.	LLMs are used directly for evidence-grounded report generation and optional judge-based audit.
Interpretation style	Human-written synthesis after graph analysis.	Automatic generation of global process reports and anchor-centric reports.

## Data Availability

The platform code and derived analysis artifacts (cached CSV/JSON outputs and prompt/output exports used for the application-layer case study) is available at https://github.com/pathology-dynamics (accessed on 19 May 2026). The SemNet 2.0 graph snapshot used in this study may be subject to licensing and redistribution constraints.

## Appendix A. Representative Exported Application-Layer Cases

This appendix provides representative exported cases from the deployed application to complement the summary in the main text. Each case was selected to illustrate variation across report family, semantic layer, and region. The complete exported artifact directory for each case contains four files: report_prompt.txt, report.txt, judge_prompt.txt, and judge.txt.

**Table A1.** Representative exported cases included in the appendix.

Case	Family	Layer	Region	Representative Focus
G1	Global	DSYN	*R*_123_	Shared neuro-psychiatric/ neurodegenerative mechanism
G2	Global	AAPP	*R*_123_	Shared protein aggregation mechanism
G3	Global	AAPP	*R*_1_	Alzheimer’s-biased energy and metabolism mechanism
G4	Global	DSYN	*R*_1_	Alzheimer’s-biased endocrine mechanism
A1	Anchor	PHSU	*R*_123_	Shared multimorbidity mechanism for *Acetylcholinesterase Inhibitors*
A2	Anchor	DSYN	*R*_123_	Shared multimorbidity mechanism for *Parkinson Disease*
A3	Anchor	AAPP	*R*_1_	Alzheimer’s-specific mechanism for *Cytochrome P-450 CYP2D6*

### Appendix A.1. Representative Global Report Cases

#### Case G1: DSYN–*R*_123_

**Setting:** Global report; DSYN layer; shared tri-disease core *R*_123_. **Excerpt:** “Neurodegenerative processes, such as those seen in Neurodegenerative Disorders and Cerebral atrophy, contribute to shared cognitive and motor impairments across Alzheimer’s Disease, Amyotrophic Lateral Sclerosis, and Frontotemporal dementia. These conditions often co-occur due to overlapping neurodegenerative mechanisms, leading to progressive brain atrophy and dementia.” **Judge scores:** Content accuracy = **80**; expression quality = **70**.

#### Case G2: AAPP–*R*_123_

**Setting:** Global report; AAPP layer; shared tri-disease core *R*_123_. **Excerpt:** “Aberrant protein aggregation, particularly involving amyloid-beta peptides, tau proteins, and alpha-synuclein, underlies neurodegeneration in Alzheimer’s Disease, Amyotrophic Lateral Sclerosis, and Frontotemporal dementia. These aggregated proteins disrupt proteostasis, induce oxidative stress, and impair neuronal function, reflecting a shared molecular mechanism across these diseases.” **Judge scores:** Content accuracy = **90**; expression quality = **85**.

#### Case G3: AAPP–*R*_1_

**Setting:** Global report; AAPP layer; Alzheimer’s-specific region *R*_1_. **Excerpt:** “Alzheimer’s Disease is characterized by dysregulation of energy metabolism and lipid processing, particularly involving Apolipoprotein E (APOE), which is central to amyloid-beta clearance and lipid transport. APOE dysfunction contributes to amyloid plaques and neuroinflammation, mechanisms less prominent in ALS and Frontotemporal dementia. Additionally, oxidative stress pathways, such as those involving cytochrome P-450 enzymes and oxidases, may exacerbate mitochondrial dysfunction and neuronal loss, which are more pronounced in Alzheimer’s Disease.” **Judge scores:** Content accuracy = **90**; expression quality = **85**.

#### Case G4: DSYN–*R*_1_

**Setting:** Global report; DSYN layer; Alzheimer’s-specific region *R*_1_. **Excerpt:** “Endocrine dysregulation in Alzheimer’s Disease may reflect chronic inflammation or metabolic changes, distinguishing it from ALS or FTD. Steroid-induced diabetes and pituitary incidentaloma suggest systemic endocrine involvement, which is less characteristic of ALS or FTD. These findings highlight Alzheimer’s Disease’s unique syndrome progression.” **Judge scores:** Content accuracy = **80**; expression quality = **90**.

### Appendix A.2. Representative Anchor-Centric Report Cases

#### Case A1: PHSU–*R*_123_

**Setting:** Anchor-centric report; PHSU layer; shared tri-disease core *R*_123_. **Selected anchor:** *Acetylcholinesterase Inhibitors*. **Excerpt:** “Acetylcholinesterase Inhibitors (AChEIs) may reflect shared disease biology across Alzheimer’s Disease, Amyotrophic Lateral Sclerosis, and Frontotemporal Dementia by targeting cholinergic pathways, which are impaired in neurodegenerative conditions. The modulation of acetylcholine levels by AChEIs can influence Amyloid-beta processing via the Amyloid Precursor Protein (APP), a shared mediator in these diseases. Additionally, AChEIs may interact with glutamatergic signaling, as seen with memantine, which is also implicated in neuroprotection across these conditions.” **Judge scores:** Content accuracy = **80**; expression quality = **90**.

#### Case A2: DSYN–*R*_123_

**Setting:** Anchor-centric report; DSYN layer; shared tri-disease core *R*_123_. **Selected anchor:** *Parkinson Disease*. **Excerpt:** “Parkinson Disease may underlie shared multimorbidity across Alzheimer’s Disease, Amyotrophic Lateral Sclerosis, and Frontotemporal dementia through disrupted post-translational protein processing, which contributes to neurodegenerative cascades involving misfolded proteins. Additionally, impaired glucose metabolism and shared gene modules further link these diseases, reflecting a common vulnerability to neurodegeneration.” **Judge scores:** Content accuracy = **80**; expression quality = **90**.

#### Case A3: AAPP–*R*_1_

**Setting:** Anchor-centric report; AAPP layer; Alzheimer’s-specific region *R*_1_. **Selected anchor:** *Cytochrome P-450 CYP2D6*. **Excerpt:** “Cytochrome P-450 CYP2D6 may underlie Alzheimer’s Disease-specific biology by modulating neuroprotective and neurotoxic pathways uniquely relevant to amyloid-beta and tau pathologies. Altered CYP2D6 activity could influence prasterone metabolism, potentially affecting neurosteroid-mediated neuroprotection. Additionally, CYP2D6’s role in xenobiotic metabolism may contribute to oxidative stress and proteotoxicity, which are more pronounced in Alzheimer’s Disease compared to Amyotrophic Lateral Sclerosis and Frontotemporal dementia.” **Judge scores:** Content accuracy = **80**; expression quality = **90**.

## Appendix B. Implementation Details for Reproducibility

### Appendix B.1. Anchor Discovery, Traversal, and Filtering

Anchor discovery was performed by bounded two-hop traversal on the SemNet 2.0 graph. For each disease endpoint *d_i_* and selected semantic layer *T*, candidate anchors were identified by incoming two-hop paths of the form *a* → *m* → *d_i_*, where *a* is an anchor candidate in semantic type *T*, *m* is an intermediate mediator, and *d_i_* is one of the three disease endpoints. The three disease endpoint CUIs were excluded from the anchor candidate set. Candidate anchors were retained if they had at least one valid two-hop connection to at least one disease.

For each anchor–disease pair, all fixed-length two-hop paths were enumerated. Paths not matching the expected five-element representation (*a*, *r*_1_, *m*, *r*_2_, *d*) were discarded. When node-type metadata were available, mediators were filtered to allowed biomedical semantic types. Edge weights were read from the graph edge-weight dictionary, and path strength was computed as the product of the two edge weights along the two-hop chain. Anchor-level path strength for a disease was then computed by summing path strengths across all valid two-hop paths linking that anchor to the disease.

Each anchor was assigned to a Venn region according to disease membership. *R*_123_ contains anchors connected to all three diseases; *R*_12_, *R*_13_, *R*_23_ contain anchors connected to exactly two diseases; and *R*_1_, *R*_2_, *R*_3_ contain anchors connected to exactly one disease. For mediator-level drill-down, the same membership logic was applied to the mediator sets associated with a selected anchor.

### Appendix B.2. Normalization and Harmonic-Mean Ranking

For each disease *d_i_*, three anchor evidence signals were computed: HeteSim relatedness, path strength, and mediator count. Path strength and mediator count were log-compressed using log(1 + *x*). Each signal was then min–max normalized to a 0–100 scale within the current candidate set. If all values for a signal were identical, positive values were mapped to 100 and zero values to 0.

For each disease, the normalized HeteSim, path-strength, and mediator-count components were combined using a strict harmonic mean. If any component was missing or below the small positive threshold used by the implementation, the disease-specific score was set to 0. This conservative rule prevents an anchor from receiving a high score when it is strong in only one evidence dimension. Region-level scores were then computed by harmonic aggregation across the diseases defining that region. This design favors anchors with balanced, multi-signal support across the relevant disease set.

### Appendix B.3. Report-Generation Prompt Templates

Report-generation prompts were implemented as two-message chat prompts consisting of a system message and a user message. Dynamic fields such as disease names, semantic layer, region identifier, evidence payload, and output schema were filled at run-time. The templates below show the fixed prompt wording used by the deployed prompt builders, with dynamic fields shown in braces.

Global process report: system message.

You are a Senior Systems~Biologist.

Output JSON only (no markdown, no extra text). Write everything in~English.

Use ONLY the provided processes and nodes. Do NOT introduce new~entities.

Write style:
- Very concise, mechanistic, reader-facing.
- Avoid over-claiming; prefer cautious language (may/could/consistent
  with).

Evidence rule:
- For EACH process: write 2-3 short sentences explaining why it could
  contribute
  to the requested shared, pairwise, or~disease-specific reporting
  objective.
- Then select representative nodes from that process node list.
- For each selected node: explain how it supports the mechanism, not merely
  a~definition.

{semantic_layer_guidance}

Global process report: user message.

{report_title}

Diseases:
- {disease_1}
- {disease_2}
- {disease_3}

INPUT:
{input_process_payload_json}

TASK:
{mode_specific_task_rule}

Return JSON only matching the schema:
{output_schema_json}

Global process report: mode-specific task rules.

base:
- If input.processes is empty: return the axes list as an empty list.
- Otherwise, for~each process in input.processes:
  1) mechanism: 2-3 short sentences explaining how this process may
  underlie,
     reflect, or~help explain the reporting~objective.

evidence_soft:
- If input.processes is empty: return the axes list as an empty list.
- Otherwise, for~each process in input.processes:
  1) mechanism: 2-3 short sentences explaining how this process may
  underlie,
     reflect, or~help explain the reporting objective.
     - May mention 1-3 nodes from process.top_nodes as supporting examples.
     - If nodes are used, integrate them naturally into the~explanation.

evidence_inline_hard:
- If input.processes is empty: return the axes list as an empty list.
- Otherwise, for~each process in input.processes:
  1) mechanism: 2-3 short sentences explaining how this process may
  underlie,
     reflect, or~help explain the reporting objective.
   - Must explicitly use 1-3 nodes from process.top_nodes as support.
   - Do not merely list node names; explain how they support
  the~mechanism.

evidence_claim_then_example:
- If input.processes is empty: return the axes list as an empty list.
- Otherwise, for~each process in input.processes:
  1) mechanism: exactly 3 short sentences.
     - Sentence 1 must state how this process may underlie, reflect,
  or~help
       explain the reporting objective.
     - Sentences 2-3 must use 1-3 nodes from process.top_nodes as concrete
  support.
     - Do not merely list node names; explain how they support the
  mechanism.

Anchor-centric report: system message.

You are a Senior Systems~Biologist.

Output JSON only (no markdown, no extra text). Write everything in~English.

Write style:
- Very concise, mechanistic, reader-facing.
- Use cautious language when needed.
- Do not write generic filler.
- {anchor_focus_line}
- {region_focus}

Mechanism writing guide for node type '{node_type}':
{semantic_layer_guidance}

Mode-specific evidence rule:
{mode_specific_evidence_rule}

Anchor-centric report: user message.

{anchor_report_title}

Diseases:
- {disease_1}
- {disease_2}
- {disease_3}

INPUT:
{input_anchor_payload_json}

TASK:
{mode_specific_anchor_task}

Return JSON only matching the schema:
{output_schema_json}

Anchor-centric report: mode-specific task rules.

base:
- Write one concise mechanism field only.
- mechanism: 2-3 short sentences explaining why {goal_text}.

evidence_soft:
- Write one concise mechanism field only.
- mechanism: 2-3 short sentences explaining why {goal_text}.
- You MAY mention 1-3 mediators as supporting examples.
- If mediators are used, integrate them naturally into the~explanation.

evidence_inline_hard:
- Write one concise mechanism field only.
- mechanism: 2-3 short sentences explaining why {goal_text}.
- You MUST explicitly use 1-3 mediators as supporting evidence.
- Do NOT merely list mediator names; explain how they support
  the~mechanism.

evidence_claim_then_example:
- Write one concise mechanism field only.
- mechanism: exactly 3 short sentences.
- Sentence 1 MUST state why {goal_text}.
- Sentences 2-3 MUST use 1-3 mediators as concrete supporting
  examples/evidence.
- Do NOT merely list mediator names; explain how they support the
  mechanism.

The templates above define the fixed prompt wording. Runtime-expanded fields include disease names, semantic layer, region identifier, structured evidence JSON, and output schema JSON. Fully expanded prompts are stored by the deployed application as exported artifacts for representative cases.

### Appendix B.4. Judge Prompt Templates

Judge prompts were also implemented as fixed two-message templates with runtime-expanded report texts, task context, and schema fields. The templates below show the fixed wording used for the main ablation judge and for the PubMed-RAG baseline comparison.

Pairwise ablation judge: system message.

You are an expert biomedical~reviewer.

You will compare two reports generated for the SAME diseases, SAME semantic
  layer,
SAME region, and~SAME~task.

Compare two separate dimensions:

Dimension 1: content_accuracy
- biological correctness
- mechanistic plausibility
- correctness of biological roles/functions
- faithfulness to the given task and~evidence

Dimension 2: expression_quality
- concreteness rather than generic wording
- groundedness rather than vague hand-waving
- appropriately bounded claims rather than~overclaiming

Decision rules:
- For EACH dimension, choose exactly one winner: A or B.
- Keep content_accuracy and expression_quality separate.
- Do not reward longer reports unless they are more accurate or clearer.
- Output JSON only.

Pairwise ablation judge: user message.

TASK CONTEXT:
{task_context}

REPORT A:
{report_a}

REPORT B:
{report_b}

Return JSON only matching the schema:
{judge_schema_json}

Disease-only PubMed-RAG versus KG judge: system message.

You are an independent biomedical~reviewer.

You will compare two comparative reports about AD, ALS, and~FTD.
One report is generated from disease-only PubMed-RAG context.
The other report is generated from SemNet KG-derived
  region/process/anchor~evidence.

Evaluate the reports on five dimensions:
1. biomedical_plausibility
2. evidence_faithfulness
3. shared_specific_distinction
4. mechanistic_specificity
5. ~comparative_clarity

For each dimension:
- assign a score from 1 to 5 to each report
- choose the better report for that dimension
- provide a brief~reason

Also choose one overall~winner.

Important:
- PubMed-RAG does not receive SemNet region labels.
- SemNet KG receives graph-derived R123/R1/R2/R3 structure, enriched
  processes,
  and representative anchors.
- Reward reports that preserve shared-core and disease-emphasized
  distinctions
  when supported by their respective evidence.
- Output JSON only.

Disease-only PubMed-RAG versus KG judge: user message.

PUBMED-RAG REPORT:
{rag_report}

SEMNET KG REPORT:
{kg_report}

RAG CONTEXT SUMMARY:
{rag_context_summary}

KG CONTEXT SUMMARY:
{kg_context_summary}

Return JSON only matching the schema:
{judge_schema_json}

### Appendix B.5. Decoding Settings and JSON Validation

Report generation was performed using an OpenAI-compatible local LLM endpoint. In the deployed application, global process reports used temperature = 0.2 and a maximum generation length of 12,000 tokens, while anchor-centric reports used temperature = 0.6 and a maximum generation length of 2048 tokens. Judge evaluation was performed through a separate OpenAI-compatible endpoint using an independent judge model. The application streams both report and judge outputs and records the exact prompt messages as debug artifacts.

All report-generation prompts requested JSON-only output. JSON validation used a permissive extraction strategy: if the model returned fenced JSON, the JSON block was extracted; otherwise, the first complete JSON object between the first opening brace and the last closing brace was parsed. Failed parses were recorded with the raw model output for audit. This validation strategy preserves malformed outputs for inspection rather than silently discarding them.

### Appendix B.6. Adaptive Prompt Policy Selection

The adaptive prompt policy was selected from the ablation summaries using a content-first rule. For each report family, semantic layer, and region, candidate evidence-grounded modes were ranked primarily by content-accuracy win rate against the base prompt. Expression-quality win rate was used as a secondary criterion. If the best evidence-grounded mode did not exceed the 0.5 content-accuracy parity threshold, the deployed policy fell back to the base prompt. This rule was applied separately for global process reports and anchor-centric reports.

The resulting policy is therefore deterministic after the ablation results have been computed. Global reports use stronger evidence grounding only in settings where content accuracy improves over the base prompt, whereas anchor-centric reports more often select mediator-grounded modes because local mediator evidence is more directly aligned with the reporting target.

### Appendix B.7. Anchor-to-Process Category Inventories and Classification Prompt

The anchor-to-process classifier used closed-set category inventories. For AAPP and related molecular layers, the micro-mechanistic ontology contained eight categories: Cell cycle regulation; Energy and metabolism; Gene regulation and expression; Inflammation and immune response; Membrane regulation; Protein aggregation; Stress response regulation; and Synapse and neurotransmission. For DSYN, PHSU, and related higher-level layers, the macro-systemic ontology contained thirteen categories: Metabolic; Immune/Inflammatory/Infectious; Cardiovascular; Neuro-Psychiatric; Sensory; Gastrointestinal; Endocrine; Hematological; Dermatological; Liver; Kidney; Musculoskeletal/Orthopedic; and Environmental Toxins.

Classification was implemented as a strict single-label task. For each node, the classifier was given the node name, CUI, and the complete category inventory, and was instructed to return exactly one category in JSON format. The first attempt requested an exact category name. If parsing failed or the returned category did not match the inventory, a fallback prompt requested the integer category ID. Outputs not matching the category inventory after retry were treated as unclassified rather than coerced into a category.

Single-label classification prompt.

System:
You are a biological category classifier. Return JSON ONLY
(no extra words, no markdown).

User:
Choose exactly ONE category name from this list (must match EXACTLY):

{category_inventory}

Entity: {node_name} (CUI:{node_cui})

Return JSON ONLY (single line):
{"category":"<ONE EXACT CATEGORY NAME FROM LIST>"}

Fallback category-ID prompt.

Choose exactly ONE category ID from this list:

{numbered_category_inventory}

Entity: {node_name} (CUI:{node_cui})

Return JSON ONLY:
{"id": <INTEGER 1-{n_categories}>}

The category inventories were inherited from prior SemNet 2.0 studies rather than defined de novo. The micro-mechanistic AAPP categories were previously used to map high-ranking AD–ALS–FTD molecular nodes to biological processes with supervised human review. The macro-systemic DSYN ontology was previously validated through independent full-text review by three human evaluators, with high inter-rater reliability (Cohen’s *κ* = 0.88). Thus, the present study uses the LLM classifier mainly to apply previously defined and human-reviewed category systems consistently across new region-level anchor outputs.

## Appendix C. Full Retrospective Validation Reports

This appendix provides the full generated SemNet Explorer reports used for the retrospective validation analysis summarized in Table 5. Reports are shown after removal of model reasoning text. For each validation case, we provide the validation target, report setting, original expert-derived finding, recovery judgment, and full generated report. These reports provide the source evidence for the qualitative recovery judgments reported in the main text.

### Appendix C.1. AD–ALS–FTD Comparative Pathophysiology

Global Report: Shared AD–ALS–FTD Molecular Overlap

**Validation target:** AD, ALS, and FTD shared molecular overlap.

**Report setting:** Global report; node type = AAPP; region = R123.

**Original expert-derived finding:** Shared inflammatory and synaptic signaling processes dominate overlap across AD, ALS, and FTD.

**Recovery judgment:** Partial recovery. The report recovered shared synaptic/neurotransmission and protein-aggregation mechanisms; inflammatory signaling was less explicit.

**Full generated report**:

{
  "report_type": "core",
  "region": "R123",
  "core_axes": [
    {
      "process": "Synapse and neurotransmission",
      "mechanism": "Dysregulation of glutamate receptors, including N-Methyl-D-Aspartate Receptors and
     AMPA Receptors, may contribute to synaptic dysfunction and excitotoxicity, which are shared
     features across Alzheimer's Disease, Amyotrophic Lateral Sclerosis, and~Frontotemporal dementia.
     This disruption could impair neurotransmission and synaptic plasticity, leading to cognitive
     decline and neuronal loss."
    },
    {
      "process": "Protein aggregation",
      "mechanism": "Aberrant aggregation of proteins such as Amyloid beta-Peptides, tau Proteins, and~ 
     alpha-Synuclein may drive neurodegeneration by disrupting proteostasis and causing synaptic
     dysfunction. These shared pathological hallmarks could underlie the progressive neuronal loss
     observed in Alzheimer's Disease, Amyotrophic Lateral Sclerosis, and~Frontotemporal dementia."
    }
  ],
  "headline": "Shared molecular mechanisms in Alzheimer's Disease, Amyotrophic Lateral Sclerosis, and~ 
     Frontotemporal dementia involve synaptic dysfunction and protein aggregation.",
  "summary": "The shared comorbidity across these neurodegenerative diseases may stem from disrupted
     neurotransmission and excitotoxicity mediated by glutamate receptor dysregulation, as~well as
     neurotoxic protein aggregation leading to synaptic and neuronal dysfunction."
}

### Appendix C.2. AD–ALS–FTD Multimorbidity

#### Appendix C.2.1. Global Report: Shared Multimorbidity Core

**Validation target:** AD, ALS, and FTD multimorbidity architecture.

**Report setting:** Global report; node type = DSYN; region = R123.

**Original expert-derived finding:** AD, ALS, and FTD share a convergent metabolic–immune multimorbidity core.

**Recovery judgment:** Partial recovery. The report recovered metabolic and neuropsychiatric disease-system organization; immune signaling was less explicit.

**Full generated report**:

{
  "report_type": "core",
  "region": "R123",
  "core_axes": [
    {
      "process": "Neuro-Psychiatric",
      "mechanism": "The shared neurodegenerative nature of Alzheimer's Disease, Amyotrophic Lateral
     Sclerosis, and~ Frontotemporal dementia underlies their comorbidity. Neurodegenerative Disorders (
     C0524851) and Tauopathies (C0949664) represent common pathological hallmarks, while Motor Neuron
     Disease (C0085084) highlights overlapping neurodegenerative pathways affecting both cognitive and
     motor systems."
    },
    {
      "process": "Metabolic",
      "mechanism": "Metabolic disturbances and multiple pathologies (C0679225) contribute to the shared
     burden of these neurodegenerative diseases. Degenerative disorder (C1285162) and metabolic
     disturbance (C0746556) reflect systemic vulnerabilities that exacerbate neurodegenerative
     progression, linking chronic disease states across Alzheimer's Disease, Amyotrophic Lateral
     Sclerosis, and~ Frontotemporal dementia."
    }
  ],
  "headline": "Neuro-Psychiatric and Metabolic Pathways Drive Shared Multimorbidity Across Alzheimer's
     Disease, Amyotrophic Lateral Sclerosis, and~Frontotemporal Dementia.",
  "summary": "The shared neurodegenerative and metabolic mechanisms underpinning Alzheimer's Disease,
     Amyotrophic Lateral Sclerosis, and~Frontotemporal dementia explain their comorbidity. Neuro-
     Psychiatric processes highlight common pathological features, while Metabolic disturbances
     exacerbate neurodegenerative progression."
}

#### Appendix C.2.2. Pairwise Global Report: AD–FTD Bridge-Related Region

**Validation target:** AD–FTD pairwise multimorbidity region involving FTD.

**Report setting:** Global report; node type = DSYN; region = R13.

**Original expert-derived finding:** FTD functions as a bridge disease in multimorbidity space.

**Recovery judgment:** Partial recovery. The report supported FTD-associated pairwise disease-system organization.

**Full generated report**:

{
  "report_type": "pairwise",
  "region": "R13",
  "headline": "Tau proteinopathy and neurodegenerative processes link Alzheimer's Disease and
     Frontotemporal Dementia, distinguishing them from Amyotrophic Lateral Sclerosis.",
  "pair_axes": [
    {
      "process": "Neurodegenerative processes",
      "mechanism": "Tau proteinopathy and neurofibrillary tangles are central to both Alzheimer's
     Disease and Frontotemporal Dementia, contributing to neuronal death and cognitive decline. These
     mechanisms are less prominent in Amyotrophic Lateral Sclerosis, which primarily involves motor
     neuron degeneration.",
      "supporting_nodes": [
        {
          "node_name": "Tau proteinopathy",
          "node_cui": "CUI1",
          "mechanistic_support": "Tau proteinopathy drives neurodegeneration in both diseases, leading
      to cognitive impairment. This mechanism is not as central in ALS, where other proteinopathies
      dominate."
        },
        {
          "node_name": "Neurofibrillary tangles",
          "node_cui": "CUI2",
          "mechanistic_support": "Neurofibrillary tangles, caused by tau accumulation, damage neurons in
      AD and FTD. This feature is not a primary hallmark in ALS."
       },
       {
          "node_name": "Neuronal death",
          "node_cui": "CUI3",
          "mechanistic_support": "Neuronal death due to tau-related damage leads to cognitive decline in
      both diseases. ALS primarily affects motor neurons through different pathways."
       }
     ]
    }
  ],
  "summary": "Alzheimer's Disease and Frontotemporal Dementia share tau-related neurodegenerative
      mechanisms, distinguishing them from Amyotrophic Lateral Sclerosis."
}

#### Appendix C.2.3. Pairwise Global Report: ALS–FTD Bridge-Related Region

**Validation target:** ALS–FTD pairwise multimorbidity region involving FTD.

**Report setting:** Global report; node type = DSYN; region = R23.

**Original expert-derived finding:** FTD functions as a bridge disease in multimorbidity space.

**Recovery judgment:** Partial recovery. The report supported FTD-associated pairwise disease-system organization.

**Full generated report**:

{
  "report_type": "pairwise",
  "region": "R23",
  "headline": "ALS and FTD share a liver-related axis involving chronic inflammation and metabolic
     dysfunction, which is not shared with Alzheimer's Disease.",
  "pair_axes": [
    {
      "process": "Liver",
      "mechanism": "Chronic liver inflammation and metabolic dysfunction may contribute to
     neurodegenerative pathways differently in ALS and FTD compared to AD, potentially through shared
     inflammatory cascades or toxin accumulation.",
      "supporting_nodes": [
        {
          "node_name": "Hepatitis C relapse",
          "node_cui": "C3160896",
          "mechanistic_support": "Hepatitis C relapse can exacerbate chronic inflammation, which may
     worsen neurodegenerative processes in ALS and FTD. This mechanism may not be as prominent in AD."
       }
     ]
    }
  ],
  "summary": "ALS and FTD share liver-related mechanisms that contribute to their pathogenesis, distinct
      from Alzheimer's Disease."
}

### Appendix C.3. Parkinson"s Disease Antihistamine Repurposing

Anchor Report: Antihistamine Therapeutic Rationale

**Validation target:** Antihistamines and Parkinson"s disease.

**Report setting:** Anchor report; anchor = Antihistamines; target = Parkinson"s disease.

**Original expert-derived finding:** Antihistamines emerged as promising repurposed adjuvant therapies for Parkinson"s disease, with mechanisms involving oxidative stress reduction, inflammatory modulation, and neurotransmitter balancing.

**Recovery judgment:** Recovered at anchor level. The report recovered histamine receptor modulation, neuroinflammation, oxidative stress, dopaminergic regulation, and neurotransmitter-related mechanisms.

**Full generated report**:

{
  "report_type": "anchor_core_report",
  "region": "R123",
  "anchor": "Antihistamines",
  "mechanism": "Antihistamines may reflect shared disease biology across Parkinson's Disease by
     targeting histamine receptors, which are involved in neuroinflammation and dopaminergic neuron
     regulation. Histamine receptor modulation can influence neuroprotective pathways, potentially
     reducing inflammation and oxidative stress. Additionally, variations in CYP2D6 gene metabolism may
      affect individual responses to antihistamines, influencing their therapeutic impact."
}

### Appendix C.4. CML TKI Adverse Events

#### Appendix C.4.1. Global Report: Shared TKI Adverse-Event Structure

**Validation target:** TKI-associated adverse-event clusters.

**Report setting:** Global report; node type = PATF; region = R123.

**Original expert-derived finding:** TKI therapy is associated with cardiometabolic, inflammatory, renal, and hematologic adverse-event clusters.

**Recovery judgment:** Partial global recovery. The report recovered shared inflammatory and metabolic adverse-event structure.

**Full generated report**:

{
  "report_type": "core",
  "region": "R123",
  "core_axes": [
    {
      "process": "Immune/Inflammatory/Infectious",
      "mechanism": "Immune dysregulation and chronic inflammation may underlie shared comorbidities
     across these diseases, such as increased infection risk and autoimmune-like symptoms. Shared
     immune-mediated pathways could explain overlapping clinical features, including fever, fatigue,
     and~organ involvement."
    },
    {
      "process": "Metabolic",
      "mechanism": "Disruption of metabolic pathways may contribute to shared clinical burden, such as
     anemia, weight loss, and~organ dysfunction. Common metabolic perturbations could underlie
     progression patterns and shared risk factors across these diseases."
    }
  ],
  "headline": "Shared immune-inflammatory and metabolic dysregulation may explain comorbidity across
     these diseases.",
  "summary": "Immune/inflammatory/infectious and metabolic processes likely contribute to shared
     clinical features and progression patterns across these diseases. These axes may underlie common
     risk factors and downstream consequences, such as infection susceptibility and organ dysfunction."
}

#### Appendix C.4.2. Anchor Report: Ponatinib Cardiovascular Adverse-Event Biology

**Validation target:** Ponatinib-specific cardiovascular adverse-event structure.

**Report setting:** Anchor report; anchor = Ponatinib.

**Original expert-derived finding:** Drug-specific adverse-event structures emerged, including ponatinib–cardiovascular events.

**Recovery judgment:** Recovered at anchor level. The report linked ponatinib to vascular, endothelial, nitric oxide, coagulation, and cardiovascular mechanisms.

**Full generated report**:

{
  "report_type": "anchor_core_report",
  "region": "R123",
  "anchor": "ponatinib",
  "mechanism": "Ponatinib, a~kinase inhibitor, may reflect shared disease biology across cardiovascular
     diseases by targeting pathways involved in inflammation, vascular tone, and~coagulation. Its
     inhibition of phosphotransferases modulates signaling pathways that contribute to endothelial
     dysfunction and atherosclerosis. Additionally, ponatinib's modulation of nitric oxide levels can
     influence vascular tone and inflammation, aligning with shared mechanisms in cardiovascular
     pathogenesis."
}

#### Appendix C.4.3. Anchor Report: Nilotinib Diabetes/Metabolic Adverse-Event Biology

**Validation target:** Nilotinib-specific diabetes/metabolic adverse-event structure.

**Report setting:** Anchor report; anchor = Nilotinib.

**Original expert-derived finding:** Drug-specific adverse-event structures emerged, including nilotinib–diabetes/metabolic events.

**Recovery judgment:** Recovered at anchor level. The report linked nilotinib to inflammatory cytokines, insulin resistance, glucose metabolism, and diabetes/metabolic biology.

**Full generated report**:

{
  "report_type": "anchor_core_report",
  "region": "R123",
  "anchor": "nilotinib",
  "mechanism": "Nilotinib, a~kinase inhibitor targeting BCR-ABL, may reflect shared disease biology in
     diabetes mellitus by modulating kinase signaling pathways involved in inflammation and insulin
     resistance. By~inhibiting interleukin-6 and interleukin-1β, nilotinib could reduce proinflammatory cytokine activity, which is linked to diabetic complications. Additionally,
     modulation of phosphorylation events by nilotinib may influence cellular processes critical to
     glucose metabolism and insulin sensitivity."
}

### Appendix C.5. Diabetic Kidney Disease Signaling

#### Appendix C.5.1. Anchor Report: VEGF Signaling

**Validation target:** DKD endothelial-inflammatory signaling; VEGF module.

**Report setting:** Anchor report; anchor = VEGF; target = diabetic nephropathy.

**Original expert-derived finding:** VEGF and endothelial permeability pathways were prominent in DKD signaling.

**Recovery judgment:** Recovered at anchor level. The report linked VEGF to endothelial, vascular, permeability, and inflammatory mechanisms.

**Full generated report**:

{
  "report_type": "anchor_core_report",
  "region": "R123",
  "anchor": "VEGF",
  "mechanism": "VEGF may underlie shared disease biology across all three diseases by promoting
     angiogenesis and vascular permeability, which are central to diabetic nephropathy pathogenesis.
     Elevated VEGF levels contribute to glomerular hyperfiltration and albuminuria, supported by
     evidence of nitric oxide dysregulation via NOS3, which exacerbates oxidative stress. Additionally,
      microRNAs modulate VEGF expression, further linking this axis to disease progression."
}

#### Appendix C.5.2. Anchor Report: NF-*κ*B Signaling

**Validation target:** DKD inflammatory signaling; NF-*κ*B module.

**Report setting:** Anchor report; anchor = NF-*κ*B; target = diabetic nephropathy.

**Original expert-derived finding:** NF-*κ*B and cytokine signaling pathways were prominent in DKD signaling.

**Recovery judgment:** Recovered at anchor level. The report linked NF-*κ*B to inflammatory, cytokine, immune, and oxidative-stress mechanisms.

**Full generated report**:

{
  "report_type": "anchor_core_report",
  "region": "R123",
  "anchor": "NF-kappa B",
  "mechanism": "NF-kappa B may underlie shared disease biology in Diabetic Nephropathy by regulating
     inflammatory and immune responses, which are central to kidney damage. Activation of NF-kappa B
     leads to increased production of pro-inflammatory cytokines, exacerbating renal inflammation.
     Additionally, NF-kappa B modulates oxidative stress pathways, contributing to glomerular and
     tubular dysfunction."
}

#### Appendix C.5.3. Anchor Report: MAPK/ERK Signaling

**Validation target:** DKD inflammatory and oxidative-stress signaling; MAPK/ERK module.

**Report setting:** Anchor report; anchor = MAPK/ERK; target = diabetic nephropathy.

**Original expert-derived finding:** ERK/MAPK signaling pathways were prominent in DKD signaling.

**Recovery judgment:** Recovered at anchor level. The report linked MAPK/ERK to inflammatory, oxidative-stress, and tissue-remodeling mechanisms.

**Full generated report**:

{
  "report_type": "anchor_core_report",
  "region": "R123",
  "anchor": "MAPK",
  "mechanism": "The MAPK pathway is central to the shared molecular mechanisms in diabetic nephropathy,
     as~it regulates inflammation, fibrosis, and~oxidative stress. ERK activation promotes podocyte
     dysfunction by increasing oxidative stress and inflammation. JNK signaling contributes to fibrosis
      by enhancing extracellular matrix deposition in the kidney."
}

#### Appendix C.5.4. Anchor Report: JAK/STAT Signaling

**Validation target:** DKD cytokine and inflammatory signaling; JAK/STAT module.

**Report setting:** Anchor report; anchor = JAK/STAT; target = diabetic nephropathy.

**Original expert-derived finding:** JAK/STAT and cytokine signaling pathways were prominent in DKD signaling.

**Recovery judgment:** Recovered at anchor level. The report linked JAK/STAT to cytokine, inflammatory, and fibrotic signaling mechanisms.

**Full generated report**:

{
  "report_type": "anchor_core_report",
  "region": "R123",
  "anchor": "JAK",
  "mechanism": "JAK signaling may underlie shared disease biology by regulating inflammation and
     fibrosis, common pathophysiological processes in diabetic nephropathy. Increased JAK activity
     could drive protein biosynthesis, contributing to extracellular matrix remodeling and kidney
     damage. Dysregulated protein production may exacerbate chronic inflammation and fibrotic
     progression in diabetic nephropathy."
}

## Abbreviations

CUI: Concept Unique Identifier (UMLS)
LLM: Large Language Model
KG: Knowledge Graph
Venn: Overlap-based region decomposition
